# MBD2 promotes B cell differentiation and BCR signaling in systemic lupus erythematosus by regulating the LEF-1-PTEN-PI3K axis

**DOI:** 10.1038/s41419-025-07750-6

**Published:** 2025-06-04

**Authors:** Yukai Jing, Yunfei Zhang, Xiaocui Wang, Yufen Wang, Ying Hu, Bin Wen, Xin Dai, Xuemei Duan, Haonan Li, Shumin Dong, Ze Yan, Yufeng Fan, Cong-yi Wang, Xiansheng Liu, Ruiping Zhang

**Affiliations:** 1 Department of Clinical Laboratory, Third Hospital of Shanxi Medical University, Shanxi Bethune Hospital, Shanxi Academy of Medical Sciences, Tongji Shanxi Hospital, Taiyuan, China; 2 Institute of Clinical Medicine, Central People’s Hospital of Zhanjiang, Zhanjiang, China; 3 Department of Respiratory and Critical Care Medicine, the Center for Biomedical Research, NHC Key Laboratory of Respiratory Diseases, Tongji Hospital, Tongji Medical College, Huazhong University of Science and Technology, Wuhan, China; 4 Department of Respiratory and Critical Care Medicine, Shanxi Bethune Hospital, Shanxi Academy of Medical Science, Tongji Shanxi Hospital, Third Hospital of Shanxi Medical University, Taiyuan, China; 5 The Radiology Department of Shanxi Provincial People’s Hospital Affiliated to Shanxi Medical University, Taiyuan, China

**Keywords:** Epigenetics in immune cells, Germinal centres

## Abstract

Systemic lupus erythematosus (SLE) is a typical autoimmune disease characterized by the overproduction of autoantibodies and type I interferon, which damages its own tissues, causing multiple organ damage. B cells are thought to play a major role in the pathogenesis of SLE. As a DNA methylation reader, Methyl-CpG-binding domain protein 2 (MBD2) has been extensively studied in the contexts of innate immunity, adaptive immunity, and autoimmune diseases. However, its specific role in B cells and SLE remains unexamined. Herein, we found that MBD2 was highly expressed in B cells of SLE patients and positively correlated with disease activity. Knockout of MBD2 in B cells disturbed B-cell differentiation, dampened B-cell activation, B-cell receptor (BCR) signaling, and T-cell-dependent humoral immune responses in mice. What’s more, MBD2 deficiency effectively attenuated lupus-like symptoms, reduced the germinal center responses, and decreased anti-dsDNA antibodies in lupus model mice. Mechanistically, MBD2 selectively bound to the methylated CpG of *Lef-1* induced by IFN-α, inhibiting the transcription and expression of *Lef-1*, which repressed *Pten* transcription and expression, thereby promoting PI3K-Akt-mTOR signaling. This study first demonstrated the role of MBD2 in the pathogenesis of SLE and provided a new target for SLE.

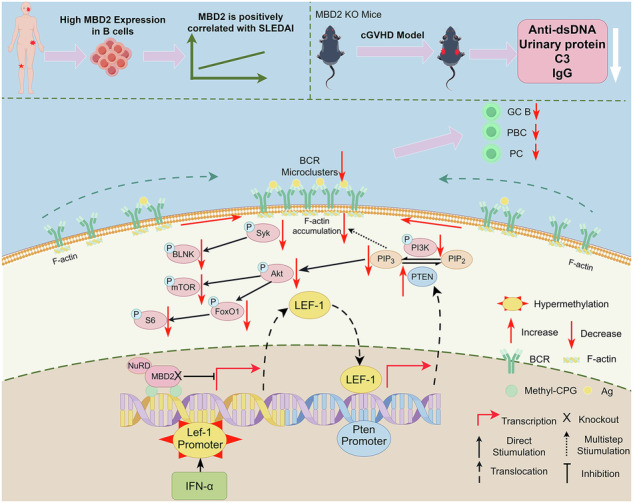

## Introduction

Systemic lupus erythematosus (SLE) is an autoimmune disease marked by the extensive generation of autoantibodies and the accumulation of antigen-antibody complexes, which leads to the damage of multiple organs, including the kidneys, the circulatory system, and the nervous system [1, 2]. The underlying cause of SLE remains elusive due to the incomplete understanding of its pathogenesis. B-cells are recognized as crucial mediators of the immune dysregulation observed in SLE, with their abnormal differentiation, development, B-cell receptor (BCR) signaling disturbances, and hyperactivity being central to the progression of disease [3, 4]. However, the molecular mechanism of B cell abnormality remains to be further revealed.

Extensive research has established a significant link between DNA methylation and the functionality of immune cells in SLE. DNA methylation, a prevalent epigenetic mechanism, entails the addition of a methyl group to the C5 position of cytosine, resulting in 5-methylcytosine [5]. Analysis of peripheral blood mononuclear cells (PBMCs) from genetically identical monozygotic twins, where one twin has SLE, showed 49 hypomethylated genomic regions in the affected twin [6]. Additionally, key cytokines involved in SLE pathogenesis, such as IFI44L, were also hypomethylated [7]. This study highlights a strong link between SLE and DNA hypomethylation, though the role of DNA hypermethylation remains unclear. Methyl-CpG-binding domain protein 2 (MBD2), a key DNA methylation reader, plays a crucial role in the DNA methylation machinery. It possesses a high affinity for methylated DNA CpG sites and facilitates chromatin structure alterations by recruiting histone deacetylases upon recognition of these methylation sites [8]. This process is instrumental in regulating gene transcription and plays a crucial role in determining the transcriptional state of the epigenome. Previous studies have uncovered the role of MBD2 in both innate and adaptive immunity, including its impact on early T-cell development [9], Th17 cell regulation [10], and macrophage differentiation and function [11]. Nonetheless, the role of MBD2 in B cells remains inadequately explored. In B cells, phosphoinositide-3-kinase (PI3K) is vital for their development, especially in maturing precursor B cells in the bone marrow [12]. Abnormal PI3K activation leads to dysregulated B cell activation and excessive antibody production in SLE [13]. However, the regulation of PI3K by DNA methylation and the role of MBD2 in lupus remain unclear.

In our study, we found that MBD2 expression in B cells from lupus patients and lupus-like mice was increased and correlated with patient disease activity. In B cell-specific MBD2 knockout mice, spontaneous germinal center (GC) differentiation, BCR signaling, early B-cell activation, and humoral immunity responses were reduced. MBD2 deficiency effectively attenuated lupus-like symptoms and reduced the GC responses in lupus mice. Mechanistically, after incubated with interferon α (IFN-α) which is increased in SLE, CpG of lymphoid enhancer-binding factor 1 (*Lef-1)* in B cells was methylated, and then MBD2 selectively bound to the CpG to inhibit the transcription and expression of *Lef-1*, which in turn inhibited the transcription and expression of *Pten*, and thus regulate PI3K-Akt-mTOR signaling then affects the pathogenic progression of SLE. Collectively, this study demonstrates the role of MBD2 in B cells and the pathogenesis of SLE, and expands the mechanism of B cell abnormalities in SLE.

## Materials and methods

### Patient information and peripheral blood mononuclear cells collection

Blood samples were obtained from individuals diagnosed with SLE and healthy counterparts. All individuals with SLE fulfilled the 1997 revised criteria for SLE established by the American College of Rheumatology. All participants were enrolled in the study after signing an informed consent form. Basic characteristics of patients and healthy controls (HC) are shown in Table S5. Clinical diagnostic data, demographic characteristics of the patients, and laboratory information were obtained from the medical record. The SLE disease activity index 2000 (SLEDAI-2K) was used to determine the SLE disease activity index (SLEDAI) score of each patient [14]. Based on the SLEDAI score, patients were divided into two groups (SLEDAI ≤ 4, inactive group; SLEDAI > 4, active group). PBMCs were isolated using Ficoll (HY2015, Tbd science) according to the instructions provided with the reagents.

### Mice

The experimental mice were aged 8–12 weeks, and *Mbd2*^fl/fl^ *Cd19*^cre/+^ mice were used as knockout (KO) mice and *Mbd2*^fl/fl^ *Cd19*^cre/+^ mice were used as wild-type (WT) controls. WT and KO mice were generated by breeding *Mbd2*^fl/+^ mice with *Cd19*^cre/+^ mice purchased from Cyagen Bioscience (Guangdong, China). WT and KO mice were all from a C57BL/6J background. Bm12 mice (B6.C-H2-Ab1bm12/KhEgJ) were obtained from the Jackson Laboratory. All mice above were housed and fed in individually ventilated cages under specific pathogen-free conditions.

### Cell isolation and purification of B cells

Mouse spleens were extracted by surgical manipulation and ground in a grinding dish to make a single-cell suspension and splenic lymphocytes were isolated by density gradient centrifugation with Ficoll (17144002, Cytiva), where T-cells were removed from splenic lymphocyte cells by complement-dependent cytotoxicity (CDC) action by the process of adding anti-CD90.2 (140302, Biolegend) and guinea pig complement (CL5000-R, Cedarlane) for 30 min at 37 °C ambient. Monocytes/macrophages were eliminated by incubation in a 37 °C, 5% CO_2_ cell incubator for 1 h to take advantage of monocyte-macrophage adherence properties [15].

### Western blot

Purified B cells were incubated with 10 μg/ml of Biotin-SP-conjugated AffiniPure F(ab’)^2^ Fragment Goat Anti-Mouse IgG+IgM (H + L) (115-066-068, Jackson ImmunoResearch) was incubated on ice for 30 min, then streptavidin (10 μg/ml) was added and incubated for another 10 min. This was followed by activation of B cells in a 37 °C water bath. Protein samples added to cell lysates were separated by SDS-PAGE and transferred to polyvinylidene fluoride membranes. After 5% skimmed milk powder was incubated for 1 h, specific protein primary antibodies were diluted with commercial primary antibody diluent and added to the corresponding protein bands. The membrane was incubated overnight at 4 °C and then washed three times with TBST for 5 min each time. The secondary antibodies were diluted with TBST containing 5% skimmed milk, washed, and then added to the target protein bands. After incubation at room temperature for 1.5 h, the membrane was washed three times with TBST, each time for 10 min. After incubation with the secondary antibody, the membrane was washed with TBST, and the immunoreactive bands were captured using the ChemiDoc™ XRS+ Imaging System (Bio-Rad), and their intensities were quantified using Image Lab software (Bio-Rad). The following specific protein primary antibodies were used: anti-Phosphotyrosine (ab10321, Abcam), anti-pSyk (2710S, Cell Signaling Technology), anti-Syk (sc-1240, Santa Cruz), anti-pBLNK (62144S, Cell Signaling Technology), anti-BLNK (sc-8003, Santa Cruz), anti-pFoxO1 (9461S, Cell Signaling Technology), anti-FoxO1 (2880S, Cell Signaling Technology), anti-PI3K p85 (phospho Y458) + PI3 kinase p55 (phospho Y199) (ab278545, Abcam), anti-PI3K (4292S, Cell Signaling Technology), anti-pAkt (sc-293125, Santa Cruz), anti-Akt (9272S, Cell Signaling Technology), anti-pS6 (4856S, Cell Signaling Technology), anti-S6 (2217S, Cell Signaling Technology), anti-pmTOR (5536S, Cell Signaling Technology), anti-mTOR (2983S, Cell Signaling Technology), anti-LEF-1 (2286S, Cell Signaling Technology) and anti-PTEN (sc-7974, Santa Cruz). β-actin was used as a protein control, anti-β-actin (66009-1-lg, Proteintech).

### Flow cytometry

Human peripheral PBMC were labeled with PE-anti-CD14 (A07764, Immunotech S.A.S), APC-anti-CD25 (302610, Biolegend), PE-Cy7-anti-CD127 (351312, Biolegend), PB-anti-CD4 (300521, Biolegend), BV510-anti-CD8 (344732, Biolegend), PE-anti-CD27 (302807, Biolegend), APC-CD19 (302212, Biolegend), Fixable Viability Stain 620 (564996, BD Pharmingen), PE-Cy7-anti-CD24 (311119, Biolegend), PB-anti-CD38 (356627, BD Pharmingen), BV510-anti-IgD (348219, Biolegend) to analyze lymphocytes. To perform intracellular staining for MBD2, the cells were fixed and permeabilized using a fixation/penetration kit (00-5523-00, eBioscience). Then cells were incubated with anti-MBD2 (BM5471, BOSTER Biological Technology) and labeled with Alexa Fluor™ 488-Goat anti-Rabbit IgG secondary antibody (R37116, Thermo Fisher Scientific).

Bone marrow (BM) cells were extracted from the tibia and femur of mice by surgical manipulation. Bone marrow cells were treated with erythrocyte lysis buffer. BM cells were then stained with FITC-anti-B220 (103206, Biolegend) or PerCP/Cyanine5.5 anti-B220 (103235, Biolegend), PE-anti-BP-1 (108307, Biolegend), 7-AAD (420403, Biolegend), APC-anti-IgM (406509, Biolegend), PE-Cy7-anti-CD43 (143209, Biolegend), Pacific Blue (PB)-CD24 (101819, Biolegend), and BV510-CD127 (135033, Biolegend). To examine the MBD2 expression in B cell subsets, Bone marrow cells were then fixed and permeabilized using a fixation/penetration kit, and then incubated with anti-MBD2 (sc-514062, Santa Cruz) and DyLight 488-Goat anti-Mouse IgG secondary antibody (BA1126, BOSTER Biological Technology).

Splenic lymphocytes obtained after Ficoll paque plus (17144002, Cytiva) density gradient centrifugation were treated with FITC anti-B220, PE anti-CD95 (152607, Biolegend), 7-AAD, AF647-anti-GL-7 (144606, Biolegend), FITC-Annexin V (640906, Biolegend), PE-anti-CD21/CD35 (123409, Biolegend), Percp/Cy5.5-anti-CD23 (101617, Biolegend), PE-anti-CD86 (105007, Biolegend), FITC-anti-CD80 (104705, Biolegend), Percp-anti-B220 (103235, Biolegend), APC-anti-CD69 (104513, Biolegend) and BV510-anti-CD138 (142521, Biolegend), BV421-anti-IgM (406517, Biolegend), PE-anti-ICOS(CD278) (313508, Biolegend), Percp-anti-CD44 (103036, Biolegend), APC-anti-CD4 (100412, Biolegend), PE-Cy7-anti-PD-1 (CD279) (109109, Biolegend), BV421-anti-CXCR5 (CD185) (145531, Biolegend) and PE-NP (N-5070-1, Biosearch) to analyze B cell peripheral differentiation for intracellular staining of PE-Cy7-anti-Ki-67 (25-5698-80, eBioScience), cells were fixed and permeabilized using the Fixation/Permeabilization Kit (00-5523-00, eBioscience).

### Confocal microscopy analysis

Purified splenic B cells were incubated with Alexa Fluor594-affinity-pure F(ab’)^2^ fragment goat anti-mouse IgG+IgM (H + L) (115-586-068, Jackson ImmunoResearch) on ice for 30 min. The cells were then activated at 37 °C for the specified durations. Afterward, the cells were fixed using 4% paraformaldehyde and permeabilized with 0.05% saponin (S4521-10G, Sigma–Aldrich). Subsequently, the cells were stained using the following antibodies: anti-phosphotyrosine pY (ab10321, Abcam), ActinGreen^TM^ 488 Probe^TM^ (R37110, Invitational), anti-pSyk (2710S, Cell Signaling Technology), anti-pBLNK (62144S, Cell Signaling Technology), and anti-pWASp (ab59278, Abcam). Finally, the cells were incubated with alexa fluor^TM^ 488 goat anti-rabbit IgG (H + L) cross-adsorbed readyprobes^TM^ (R37116, Thermofisher) secondary antibody, alexa fluor^TM^ 647 goat anti-rabbit IgG (H + L) cross-adsorbed (A-21244, Thermofisher) secondary antibody, and DyLight 488 conjugated affinipure goat anti-mouse IgG (H + L) (BA1126, Boster). The images were captured using a confocal microscope (Olympus, FV3000).

### Establishment of the lupus model

cGVHD model: Spleens and lymph nodes (mandibular, axillary, inguinal) were aseptically collected from 8 to 10-week-old female Bm12 mice. Lymphocytes were then isolated from these tissues and subsequently combined. The mixed cells were then suspended in serum-free PBS, adjusted to a concentration of 5 × 10^7^/mL, and chilled on ice until needed. On day 0 and 14, 1 × 10^7^ lymphocytes per mouse were intravenously administered into the tail veins of 6 to 8-week-old female WT and KO mice. By day 28 post-transplantation, the mice were euthanized for the collection and analysis of serum, urine, and spleen [16]. CGP049090 (C274667-5mg, Aladdin) was selected as the LEF-1 inhibitor, with treatment initiation timed to day 14 post-cGVHD induction. The dosing regimen comprised seven intraperitoneal injections of 2 mg/kg/dose, administered at two-day intervals [17]. Serum, urine, and spleen were performed on day 28 post-treatment initiation.

### LEF-1 inhibition assay in *vitro*

Following the purification of splenic B cells from WT and KO mice, the KO group was stratified into two distinct groups. One group was administered the 5 μM LEF-1 inhibitor CGP049090, while the other two groups remained untreated and were cultured for a duration of two days at 37 °C within a 5% CO_2_ incubator.

### PTEN inhibition assay in *vitro*

Following the purification of splenic B cells from WT and KO mice, the KO group was stratified into two distinct groups. One group was administered the 30 nM PTEN inhibitor BpV (HOpic) (A189911-5mg, AmBeed), while the other two groups remained untreated and were cultured for a duration of 24 h at 37 °C within a 5% CO_2_ incubator.

### T cell-dependent humoral immunity model

A mixture of 4-Hydroxy-3-nitrophenylacetyl-Keyhole Limpet Hemocyanin (NP-KLH) (N-5060-5, BioSearch) and adjuvant (77161, ThermoFisher) at a concentration of 0.2 mg/ml was prepared according to the available literature. Subsequently, WT and KO mice at 8–10 weeks of age received 200 µL of the mixture by subcutaneous injection. Twenty-eight days after the first immunization, the mice were re-vaccinated with the same dose. Finally, spleen and serum were collected thirty-three days after the first immunization.

### Enzyme-linked immunosorbent assay (ELISA)

The blood of mice after modeling was extracted, and the supernatant was centrifuged to obtain the serum. Immunoglobulins in serum were quantified by enzyme-linked immunosorbent assay. First, ELISA plate strips were covered with NP_2_-BSA and NP_30_-BSA (2 μg/ml, Biosearch) coatings. Afterwards, plate strips were covered with 10% bovine serum albumin (BSA), and mouse serum was diluted according to the indicated titers and added to the plate strips. Subsequently, HRP-conjugated goat anti-mouse IgG, Fcγ subclass 1 specific (SA00012-1, Proteintech)/HRP-conjugated goat anti-mouse IgM, μ chain specific (SA00012-6, Proteintech) secondary antibody was added, followed by the addition of substrate. Calf thymus DNA covered slides (T13592, Targetmol) were used for determining the level of anti-dsDNA in serum, followed by the addition of diluted mouse serum as well as specific secondary antibodies, the addition of substrate to develop the color, and the absorbance of OD 450 was measured.

### Immunofluorescence analysis

Spleen and kidney samples from WT mice and KO mice were embedded using optimal cutting temperature compound and cryosectioned. Sections were covered with acetone for 5 min and dried for subsequent manipulation. Spleen and kidney sections were blocked with a solution containing 5% bovine serum albumin (BSA) and 10% Fc blocker (101319, Biolegend). Sections were then stained overnight at 4 °C with the following antibodies: AF647-anti-GL7 (144606, Biolegend), Pacific Blue-anti-CD4 (100531, Biolegend), FITC-anti-CD45R/B220 (103206, Biolegend), DyLight 488-conjugated affinity-pure goat anti-mouse IgG (H + L) (BA1126, Boster), and AF488-anti-C3 (sc-58926, SantaCruz). Images were captured using a confocal microscope (Olympus, FV3000), and data were analyzed using FV31 ASW software.

### Gene ontology enrichment analysis

We identified methylation-associated genes in SLE B cells from a published supplementary dataset [18] (Table S3 in original literature), partially sourced from the GSE59520 series accessible through the Gene Expression Omnibus (GEO) database (accession: GSE59520). Thirty-eight hypermethylated genes were selected from this dataset and functionally annotated using Metascape (https://metascape.org).

### Urea, creatinine and urine protein measure

Creatinine levels were measured using the creatinine content assay kit (RXWB0459-96, RXWB Technology), respectively, following the instructions of the manufacturer. Urinary protein levels from collected urine samples were determined using a micro total protein assay kit from Desai Diagnostic Systems (Shanghai).

### Plasmid construction

*Lef-1* WT promoter was synthesized from Tsingke Biotechnology and then inserted into the pGL3-basic vector. Mutations of the *Lef-1* promoter were obtained by site-directed mutagenesis. Primers were synthesized by Tsingke Biotechnology (Beijing, China). The primers used for PCR amplification are shown in Table S1 in the Supplementary Material. All PCR reactions were performed with PrimeSTAR HS DNA Polymerase (R045B) from TAKARA. Isopropanol precipitation was used to precipitate the PCR products, which were then treated with 1.5 μL of DpnI (R0176L) from NEB with incubation at 37 °C for 2 h. Next, 3 μL of digested PCR products were transformed into 100 μL competent cells by the heat shock method. Mouse *Mbd2* were cloned from cDNA of MEF cells by PCR, and then was inserted into the pCMV expression vector.

### Real-time quantitative PCR (RT-qPCR) analysis

Total RNA was extracted using Total RNA Extraction Reagent (SM129-02, Sevenbio), and cDNA synthesis was performed using the Reverse Transcription Kit (with dsDNase) (MF166-Plus-01, Mei5bio). The extracted cDNA was then used for mRNA expression detection in WT and KO mice splenic B cells using the Rapid Fluorescence Quantitative PCR Kit (SYBR Green) (SM133-01, Sevenbio). Relative expression was quantified using the 2^−ΔΔCT^ method. The primer sequences (BioSune Biotechnology) are provided in Table S2.

### Chromatin immunoprecipitation (ChIP) assay

ChIP assays were performed using a ChIP assay kit (9003S, Cell Signaling Technology). Briefly, 1 × 10^7^ splenic B cells from WT mice were cross-linked with formaldehyde, followed by chromatin fragmentation according to the provided protocol. The diluted soluble chromatin was incubated with MBD2 and LEF-1 antibodies overnight at 4 °C with rotation. Normal rabbit IgG was used to control for non-specific binding. The mixtures were then incubated with protein A + G agarose beads, and after washing, the protein-DNA complexes were eluted. The eluted DNA was analyzed via ChIP-PCR using the indicated primers (details in Table S2).

### DNA methylation analysis of the *Lef-1* gene core promoter

The core promoter genomic DNA of the *Lef-1* gene isolated from various tissues underwent DNA methylation analysis using the DNeasy Tissue Kit (69504, Qiagen). One milligram of genomic DNA was processed using the EZ DNA Methylation-Direct Kit from Zymo for bisulfite conversion. PCR amplification of bisulfite-treated DNA was performed using specific primers (Table S3 for details) targeting CpG sites within the CpG islands of the *lef-1* core promoter. MethPrimer 2.0 (https://www.urogene.org/methprimer2/) was employed to design specific primers and predict CpG islands. The PCR products were then purified through a 1% agarose gel with the help of the Rapid Gel Extraction Kit of Qiagen, ligated into a pGEM-T vector (A1360, Promega), and introduced into TOP 10 competent bacteria (C404003, Invitrogen) for cloning. For sequencing, three colonies, each representing a single allele, were randomly chosen from every sample.

### Cell culture and transfection

HEK293T cells were purchased from American type culture collection (ATCC). HEK293T cells were cultured in DMEM supplemented with 10% FBS and 1% penicillin–streptomycin solution at 37 °C with 5% CO_2_. Transient transfection of plasmid in HEK293T cells was performed using polyethylenimine according to the manufacturer’s protocol. Cells were analyzed 48 h after transfection.

### Dual-luciferase reporter assay

HEK293T cells were plated in a 12-well plate and were transfected with a promoter with or without transcription factor; all cells were transfected with Renilla as a control. For MBD2 regulates *LEF-1* expression, pGL3-*LEF-1* WT promoter or pGL3-*LEF-1* mutation promoter was transfected with or without MBD2. For LEF-1 regulates *PTEN* expression, pGL3-*PTEN* promoter was transfected with or without LEF-1. After 48 h, the cells were collected to analyze luciferase activity using the Dual Luciferase Reporter Gene Assay Kit (11401ES60, Yeasen Biotechnology).

### Electrophoretic mobility shift assay

HEK293T cells were plated in a 10-well plate and were transfected with pCMV-LEF-1 or pCMV-MBD2. After 48 h, the cells were collected to extract nuclear protein using the Nuclear and Cytoplasmic Protein Extraction Kit (P0027, Beyotime). Biotin was used to label double-stranded DNA probes using a DNA labeling kit (GS008, Beyotime). Refer to Table S4 for Probe sequences. The EMSA/Gel-Shift EMSA Kit (GS002, Beyotime) was used for the shift assay according to the manufacturer’s instructions. DNA was detected using the Chemiluminescent EMSA Kit (GS009, Beyotime).

### Statistical analysis

Data analysis was conducted using Prism 9.5 software (GraphPad Software, La Jolla, Calif). Statistical differences between the two groups were assessed using an unpaired two-tailed Student’s *t-test* when data followed a normal distribution. For multiple comparisons, one-way ANOVA was employed. Data were presented as mean ± SEM (standard error of the mean). Correlation analyses were performed using the Pearson’s correlation coefficient (*r*). Values of *P* < 0.05 were considered statistically significant.

## Results

### The expression of MBD2 is increased in B cells from SLE and correlated with disease activity

**Fig. 1:**
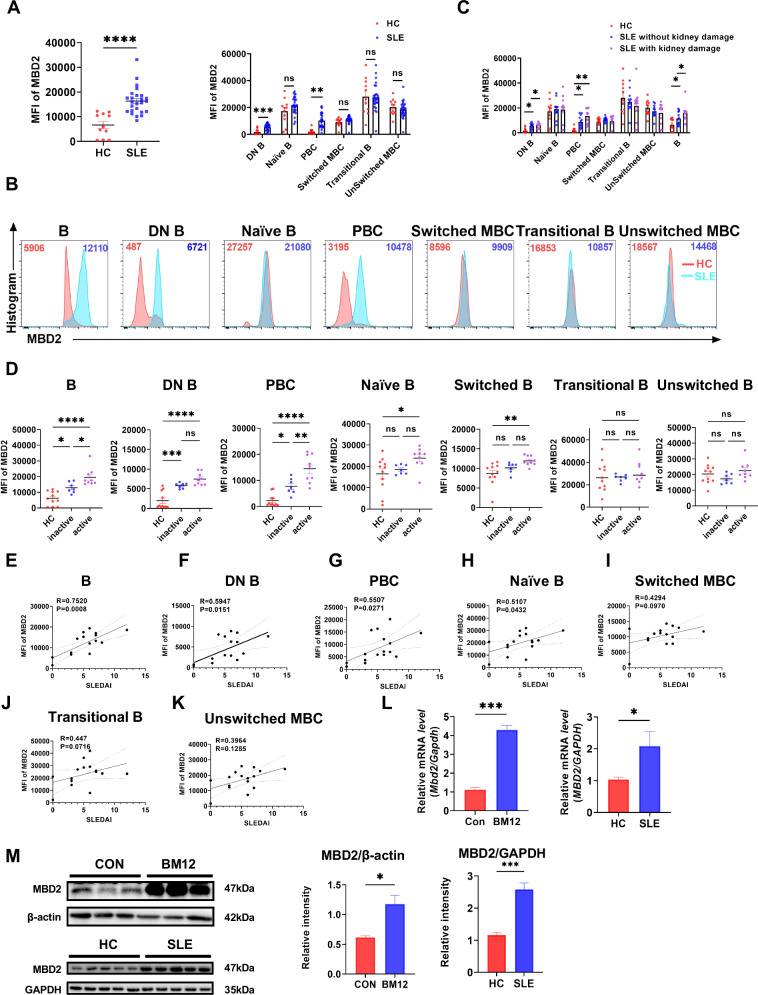
The expression of MBD2 is increased in B cells from SLE and correlated with disease activity. **A** Expression of MBD2 in peripheral blood B cell subsets from SLE patients (*n* = 25) and healthy controls (*n* = 11). **B** Flow representative plot of MBD2 in peripheral blood B cell subpopulations in SLE patients (*n* = 25) and healthy controls (*n* = 11). **C** MBD2 levels in B-cell subsets comparing SLE non-renal injury patients (*n* = 13), renal injury cases (*n* = 12) and healthy controls (*n* = 11). **D** Expression of MBD2 in B-cell subsets among inactive SLE (SLEDAI ≤ 4) (*n* = 7), active SLE (SLEDAI > 4) (*n* = 9), and healthy controls (*n* = 11). **E**–**K** Correlation between MBD2 expression and SLEDAI in B-cell subsets from SLE patients. **L** RT-qPCR of *MBD2(Mbd2)* in splenic B cells from control and Bm12 mice (*n* = 3) and in peripheral B cells obtained from SLE patients versus healthy donors (*n* = 5). **M** Immunoblot analysis of MBD2 expression in B cells from SLE patients and in splenic B cells from Bm12 mice. *P < 0.05, **P < 0.01, ***P < 0.001, ****P < 0.0001.

To determine whether MBD2 is involved in the pathogenesis SLE through its influence on B cells, flow cytometry was employed to assess MBD2 expression in peripheral circulating B cell subsets of patients with SLE and HC (Figs. 1A, B and S1A). The analysis revealed an elevation in mean fluorescence intensity (MFI) of MBD2 in peripheral blood B cells from SLE patients compared to HC. Moreover, MBD2 expression was markedly elevated in double-negative B cells (DN B, CD19^+^IgD^−^CD27^−^) and plasmablast cells (PBC, CD19^+^CD24^−^CD38^+^). In contrast, no significant differences were observed in other B cell subpopulations, including unswitched memory B cells (MBC) (CD19^+^IgD^+^CD27^+^), naïve B cells (CD19^+^IgD^+^CD27^−^), transitional B cells (CD19^+^CD38^+^CD24^+^), and switched MBC (CD19^+^IgD^−^CD27^+^). Notably, the MFI of MBD2 was higher in total B cells, DN B, and PBC in SLE patients with renal injury compared to those without renal injury and HC (Fig. 1C) [6]. These results imply that MBD2 may contribute to the pathogenesis of SLE via B cells.

To further examine the impact of MBD2 expression on SLE disease activity, patients were categorized into inactive (SLEDAI ≤ 4) and active groups (SLEDAI > 4) according to the SLEDAI-2K. The active group exhibited higher MFI of MBD2 in B cells and PBC than the inactive group. The remaining B cell subsets, including DN B cells, naïve B cells, unswitched MBC, transitional B cells, and switched MBC, demonstrated no statistically significant differences (Fig. 1D). Further correlation analysis demonstrated a significant positive correlation between SLEDAI and MBD2 MFI in B cells, DN B cells, PBC, and naïve B cells (Fig. 1E–H), while there was no significant correlation observed in other B cell subsets (Fig. 1I–K). In addition, MBD2 mRNA and protein expression levels were increased in peripheral blood B cells of lupus patients and splenic B cells of cGVHD model mice (Fig. 1L, M) (referred to as the Bm12 model). These findings suggest that MBD2 is involved in the development and progression of SLE by affecting B cells.

### MBD2 deficiency disrupts the differentiation of B cells

**Fig. 2:**
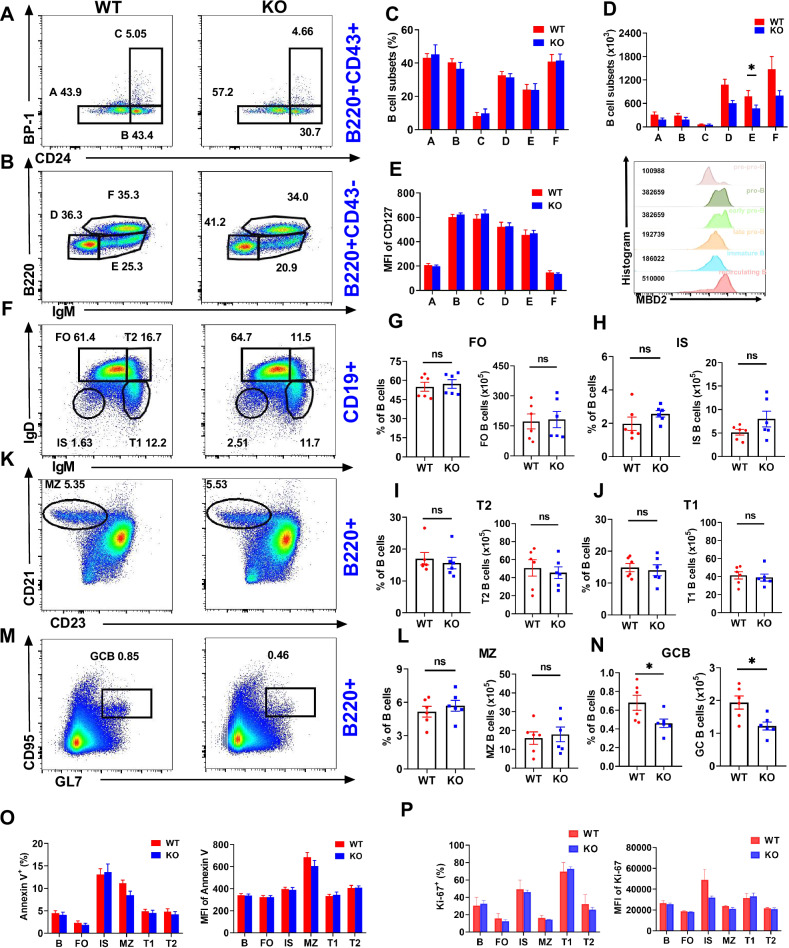
MBD2 deficiency disrupts the B cells differentiation. **A**–**F** Flow cytometry and analysis of pre pro-B (**A**), pro-B (**B**), early pre-B (**C**), late pre-B (**D**), immature B (**E**) and recirculating mature B (**F**) cells in bone marrow from WT (*n* = 6) and KO mice (*n* = 6). **E** (Left) Statistical analysis of the MFI of CD127 in bone marrow B cell subsets of WT and KO mice; (Right) MFI of MBD2 in bone marrow B cell subsets of WT mice. **F**–**L** Flow cytometry and analysis of FO, T1, T2, IS, and MZ B cells in spleens from WT (*n* = 6) and KO mice (*n* = 6). **M**, **N** Flow cytometry and analysis of GC B cells in spleens from WT (*n* = 6) and KO mice (*n* = 6). **O**, **P** Analysis proportions of apoptotic (Annexin V) and proliferative (Ki-67) in B cells, FO, IS, MZ, T1, and T2 B cells in spleens from WT (*n* = 6) and KO mice (*n* = 6); MFI of Annexin V and Ki-67 are shown. *P < 0.05.

Since abnormal development and differentiation of B cells play a crucial role in the pathogenesis of SLE [19], we hypothesized that the knockdown of MBD2 could potentially impact the development and differentiation of B cells. To address this hypothesis, we generated *Mbd2*^fl/fl^ *Cd19*^+/+^ (WT) and *Mbd2*^fl/fl^ *Cd19*^cre/+^ (KO) mice. There were no statistically significant differences in the proportions of pre-pro (A, B220^+^CD43^+^BP-1^−^CD24^−^), pro (B, B220^+^CD43^+^BP-1^−^CD24^+^), early-pre (C, B220^+^CD43^+^BP-1^+^CD24^+^), late-pre (D, B220^+^CD43^−^IgM^−^), immature (E, B220^int^CD43^−^IgM^+^), and recirculating mature (F, B220^hi^CD43^−^IgM^+^) B cells in the bone marrow in KO mice, compared to WT mice [20](Fig. 2A–C). However, the number of immature B cells was reduced in KO mice, while no differences were observed in other B cell subsets (Fig. 2D). We examined the expression of MBD2 in B cell subpopulations using flow cytometry, and found that MBD2 was expressed in all B cell subsets in bone marrow of WT mice (Fig. 2E right). Analysis using gating strategies in Fig S1B. CD127 (IL-7R), which had participated in the survival, proliferation, and maturation of bone marrow B cells [21, 22], revealed no notable difference between WT and KO (Fig. 2E left). These findings suggest that deletion of MBD2 may not affect the B cell development. Further exploration into splenic B cells, including follicular (FO, CD19^+^IgM^lo^IgD^hi^), transplantation type I (T1, CD19^+^IgM^hi^IgD^lo^) and II (T2, CD19^+^IgM^hi^IgD^hi^), isotype-switching (IS, CD19^+^IgM^-^IgD^-^), marginal zone (MZ, B220^+^CD21^hi^CD23^low^), and spontaneous GC (GCB, B220^+^GL7^+^CD95^hi^) B cells, showed no differences in the proportions and numbers of these cells between WT and KO mice [23], except for a significant reduction in GC B cells in the KO group (Fig. 2F–N). The GC represents a specialized microenvironment wherein B cells sequentially undergo clonal expansion, followed by somatic hypermutation (SHM) and affinity-driven antibody maturation. These dynamic processes are critically dependent on epigenetic regulatory mechanisms, particularly DNA methylation [24]. This mechanistic link underscores the likely involvement of MBD2 in orchestrating GC B cell development. Besides, no differences in cell proliferation and apoptosis were detected in these subpopulations between WT and KO mice (Fig. 2O, P; Fig. S2A). Collectively, these results indicate that MBD2 plays an essential role in B cell differentiation.

### Deficiency of MBD2 compromises B-cell activation and BCR signaling

**Fig. 3:**
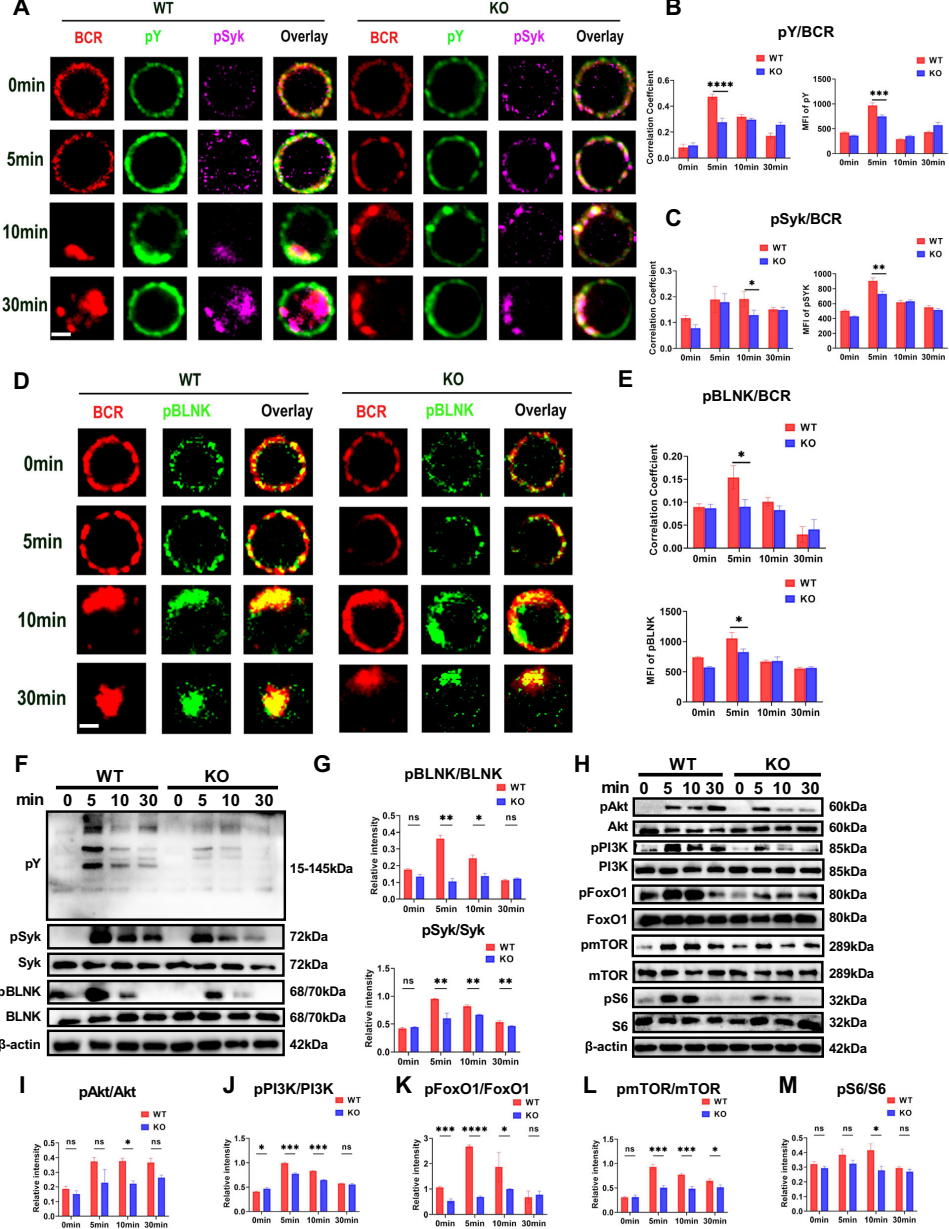
Deficiency of MBD2 compromises B cell activation and BCR signaling. **A**–**C** Confocal analysis of pY and pSyk in splenic B cells from WT and KO mice (scale bar, 2 μm). Representative plots and Pearson correlations between BCR and pY, BCR and pSyk (*n* ≥ 50 cells). **D**, **E** Confocal analysis of pBLNK in splenic B cells from WT and KO mice (scale bar, 2 μm). Representative plots and Pearson correlations between BCR and pBLNK (*n* ≥ 50 cells). **F**–**M** Immunoblot analysis of pBLNK, pSyk, pY, pPI3K, pmTOR, pAkt, pFoxO1, and pS6 in splenic B cells from WT and KO mice, with representative plots from three independent experiments. *P < 0.05; **P < 0.01; ***P < 0.001; ****P < 0.0001.

Research has shown that disrupted BCR signaling significantly contributes to the development of SLE and its immunological manifestations, while also playing a critical role in regulating B cell development and differentiation [25]. Disrupted BCR signaling, leading to a hyperreactive state, was uncovered in a recent study of a lupus mouse model [26]. To further investigate the role of MBD2 in B cell activation and BCR signaling, we examined BCR signaling in splenic B cells, which were stimulated for specific durations using soluble antigen (sAg)-F(ab′)^2^ Fragment Goat Anti-Mouse IgG+IgM (H + L). Following sAg stimulation, BCR signaling complexes on WT mice B cells underwent progressive spatial translocation from the plasma membrane to early endosomes within 5–30 min. In contrast, splenic B cells from KO mice exhibited delayed translocation kinetics, with BCR complexes accumulating only after 10–30 min post-stimulation, a timeframe significantly prolonged compared to WT counterparts (Fig. 3A). Statistical results showed that the phosphorylated tyrosine (pY), phosphorylated Syk (pSyk), and phosphorylated B cell linker protein (pBLNK) were significantly reduced at 5 min in KO splenic B cells. Additionally, the colocalization coefficients of pY, pBLNK, pSyk with BCR were markedly lower in KO mouse at both 5 and 10 min (Fig. 3A–E). Immunoblot analysis further confirmed that reductions existed in pY, pBLNK, pSyk, pPI3K, and downstream signaling molecules—such as pAkt, pmTOR, pS6, and pFoxO1 at various time points in KO mice B cells (Fig. 3F–M). These results suggest that MBD2 plays a crucial role in B cell activation and BCR signaling.

### Deficiency of MBD2 compromises B cell activation and BCR signaling by up-regulating PTEN expression

**Fig. 4:**
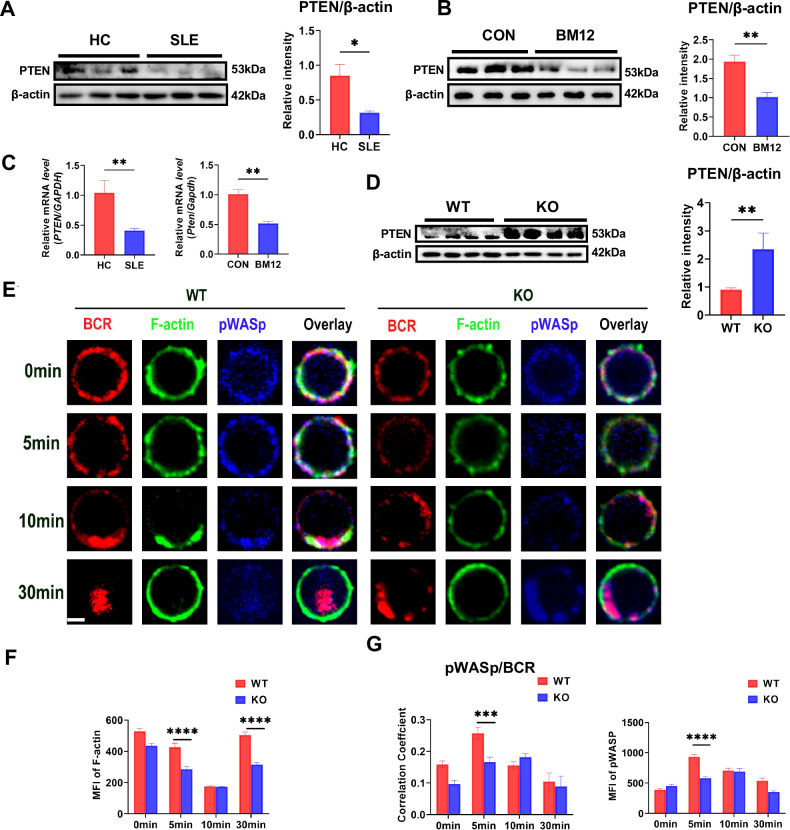
Deficiency of MBD2 compromises B cell activation and BCR signaling by upregulating PTEN expression. **A** Immunoblot analysis of PTEN expression in peripheral B cells from SLE patients (*n* = 3) and healthy controls (*n* = 3). **B** Immunoblot analysis of PTEN in splenic B cells from Bm12-induced lupus model mice (*n* = 3) and control mice (*n* = 3). **C** RT-qPCR of *PTEN* (*Pten*) mRNA in B cells from SLE patients (*n* = 6), healthy controls (*n* = 3), Bm12-induced lupus model mice (*n* = 3), and control mice (*n* = 3). **D** Immunoblot analysis of PTEN in splenic B cells from WT (*n* = 4) and KO mice (*n* = 4). **E** Confocal microscopy of F-actin and pWASp in splenic B cells from WT and KO mice (scale bar, 2 μm). **F** Quantification of MFI for F-actin splenic B cells from WT and KO mice. **G** Pearson correlations between BCR and pWASp and quantification of MFI for pWASp in splenic B cells from WT and KO mice (*n* ≥ 50 cells). Significance indicators are denoted as follows: *P < 0.05, **P < 0.01, ***P < 0.001, ****P < 0.0001.

The role of PI3K/Akt signaling in BCR signaling is recognized as crucial for B cell activation [12, 27, 28], and PTEN is recognized as a negatively factor that regulates the PI3K/Akt signaling pathway by dephosphorylating phosphatidylinositol-3,4, 5-triphosphate (PIP3) to phosphatidylinositol-4, 5-diphosphate (PIP2) [29, 30]. We found that PTEN expression was reduced in B cells from the peripheral blood of lupus patients (Fig. 4A) and from the spleen of cGVHD mice (Fig. 4B). Additionally, *PTEN* mRNA levels were consistent with protein expression (Fig. 4C). Meanwhile, PTEN significantly increase in KO splenic B cells compared to WT counterparts (Fig. 4D). In subsequent in *vitro* experiments, PTEN expression was reduced and BCR signaling, such as pBLNK, pSyk, pPI3K, pFoxO1, pmTOR, pAkt and pS6, was restored in KO mice splenic B cells after the addition of PTEN inhibitors (Fig. S2B). Thus, we concluded that MBD2 contributes to the pathogenesis of SLE by promoting BCR signaling through the suppression of PTEN expression. Previous studies have shown that PTEN inhibits DOCK2-mediated filamentous actin (F-actin) cytoskeletal reorganization by suppressing the conversion of PIP2 to PIP3, reducing BCR microcluster aggregation, and ultimately inhibiting B cell activation [31]. Wiskott-aldrich syndrome protein (WASp) was reported to regulate BCR microcluster aggregation by regulating actin reorganization [32]. We found that the MFI of F-actin was significantly reduced in KO mice B cells compared to WT mice at 5 min and 30 min following sAg stimulation (Fig. 4E, F). Additionally, MFI of pWASp and the co-localization coefficients between pWASp and BCR in KO B cells were significantly reduced at 5 min compared with WT mice (Fig. 4E, G). Therefore, these results suggest that MBD2 promotes actin polymerization to regulate early B cell activation and BCR signaling by inhibiting PTEN expression.

### Deficiency of MBD2 diminishes the humoral immune response and hinders the germinal center response

**Fig. 5:**
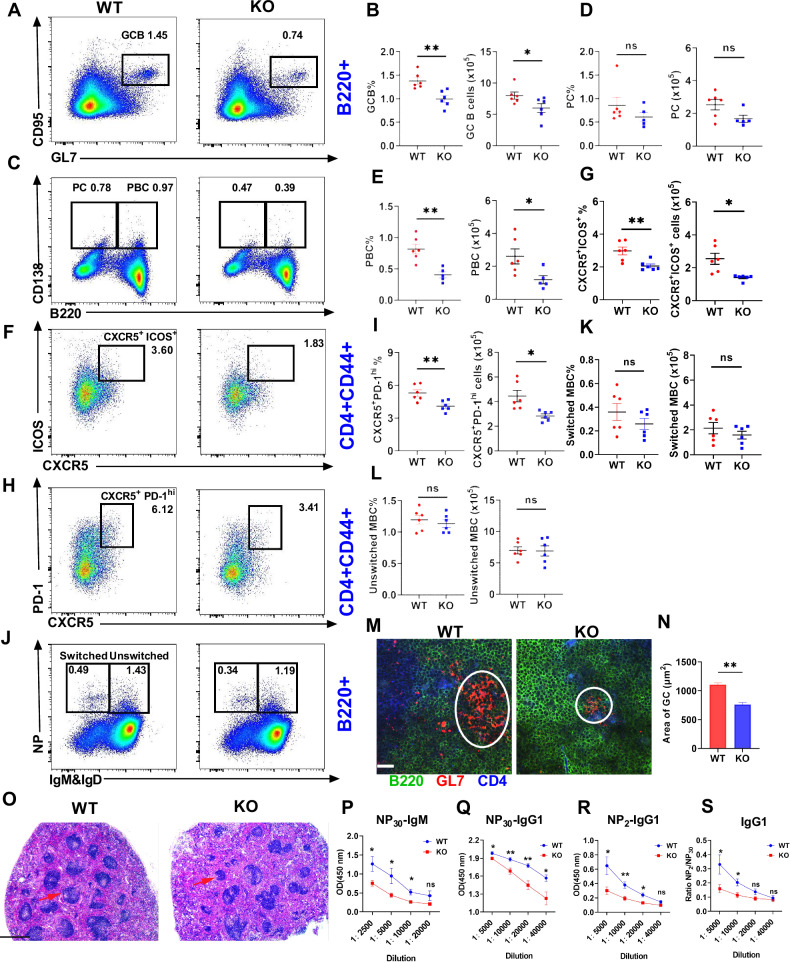
Deficiency in MBD2 attenuates the T-cell-dependent immune response. **A**–**L** Flow cytometry and analysis of GCB, PC, PBC, Tfh cells, NP-switched and unswitched memory B cells in spleens from NP-KLH immunized WT (*n* = 6) and KO mice (*n* = 6). **M** Immunofluorescence of splenic sections from immunized WT and KO mice showing GCs with anti-CD4 (blue), anti-B220 (green), and anti-GL-7 (red) antibodies (scale bar, 20 μm). **N** Quantification of GCB cell area for spatial distribution analysis. **O** HE staining of spleens from immunized WT and KO mice (scale bar, 60 μm). **P**–**S** ELISA was used to analyze levels of NP_30_-IgM, NP_30_-IgG1, and NP_2_-IgG1 in serum from immunized WT (*n* = 6) and KO mice (*n* = 6). Significance indicators are denoted as follows: *P < 0.05; **P < 0.01.

To investigate the impact of MBD2 on T cell-dependent humoral immunity, WT and KO mice were immunized with NP-KLH. Flow cytometry revealed markedly reduced frequencies of GCB, PBC (B220^+^CD138^+^), PC (B220^−^CD138^+^), and Tfh cells (CD4^+^CD44^+^CXCR5^+^ICOS^+^/PD-1^hi^) in KO mice splenic B cells compared to WT at day 14 post-immunization (Fig. 5A–I). Conversely, there were no significant differences in number and percentages of other cell subsets, such as NP-switched and unswitched memory B cells (Fig. 5J–L and Fig. S3A–H). Histological analysis demonstrated diminished GC areas and disrupted splenic follicular architecture in KO mice (Fig. 5M–O), paralleled by reduced serum levels of NP_30_-specific IgM and IgG1 (Fig. 5P–R). Antibody affinity, assessed by NP_2_/NP_30_ IgG1 ratios, was also significantly impaired in mutants (Fig. 5S). These findings indicate that MBD2 is critical for GC maintenance, Tfh cell differentiation, and high-affinity antibody production, underscoring its role in epigenetic regulation of adaptive immunity.

### MBD2 deficiency attenuates lupus symptoms in cGVHD model mice

**Fig. 6:**
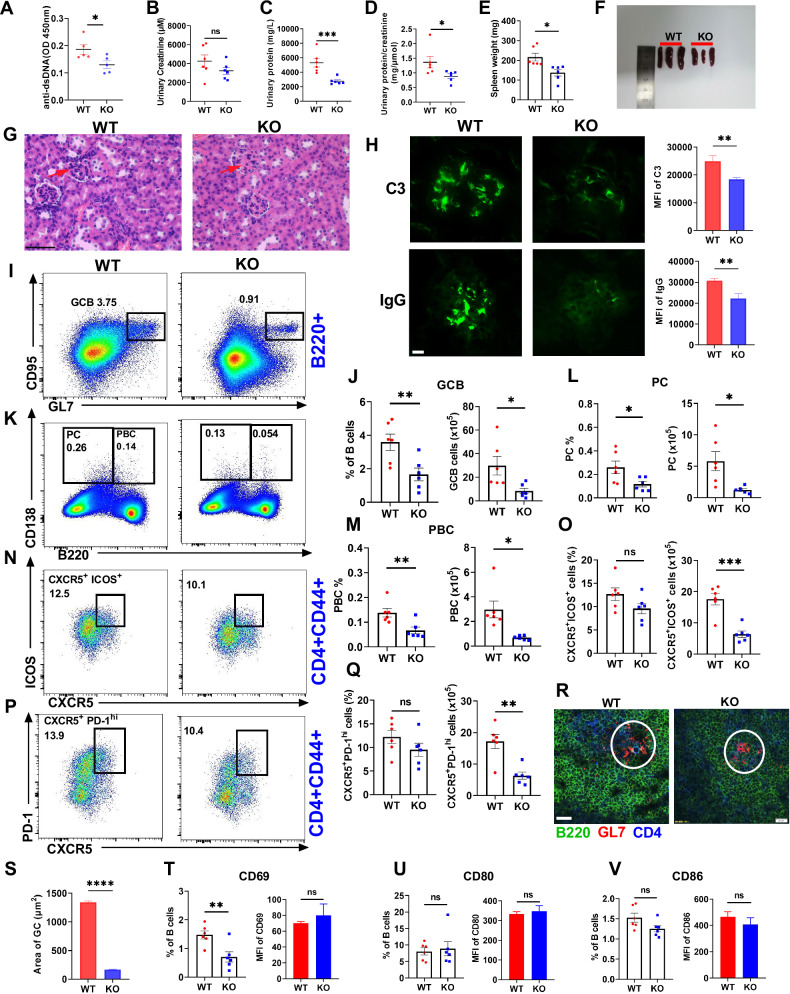
MBD2 deficiency ameliorates lupus-like autoimmunity and renal injury in a cGVHD model. **A** ELISA was used to analysis anti-dsDNA antibody in serum from Bm12-induced WT (*n* = 5) and KO mice (*n* = 5) for 4 weeks. **B** Urinary creatinine levels in Bm12-induced WT (*n* = 6) and KO mice (*n* = 6). **C** Urinary protein analysis in Bm12-induced WT (*n* = 6) and KO mice (*n* = 6). **D** Ratio of urinary protein to urinary creatinine in Bm12-induced WT (*n* = 6) and KO mice (*n* = 6). **E** Spleen weight in Bm12-induced WT (*n* = 6) and KO mice (*n* = 6). **F** Spleen size comparison between Bm12-induced WT and KO mice. **G** HE staining of kidneys from Bm12-induced WT and KO mice (scale bar, 60 μm). **H** Immunofluorescence of C3 and IgG in kidneys from Bm12-induced WT and KO mice (scale bar, 10 μm). **I**–**Q** Flow cytometry and analysis of GCB, PBC, PC, and Tfh cells in Bm12-induced WT (*n* = 6) and KO mice (*n* = 6). **R**, **S** Immunofluorescence of GCs in spleens from Bm12-induced WT and KO mice, with anti-CD4 (blue), anti-B220 (green), and anti-GL-7 (red) (scale bar, 20 μm). **T**–**V** Flow cytometry analysis of proportions and MFI of CD80, CD86, and CD69 in splenic B cells from Bm12-induced WT (*n* = 6) and KO mice (*n* = 6). *P < 0.05, **P < 0.01, ***P < 0.001, ****P < 0.0001.

To investigate the role of MBD2 deficiency in SLE progression, a cGVHD model mouse was established by transferring lymphocyte from Bm12 mice into both WT and KO mice via tail vein injection. KO mice exhibited markedly reduced serum anti-dsDNA antibody and attenuated renal injury, as evidenced by lower urinary protein levels and urinary protein/creatinine ratios compared to WT mice (Fig. 6A–D). Splenomegaly, a hallmark of lupus-like autoimmunity, was significantly alleviated in KO mice, with reduced spleen weights and spleen/total weight indices (Fig. 6E, F, S4H). Renal tissues showed reduced cellular infiltration in the glomeruli of KO mice (Fig. 6G) and fewer complement C3 and IgG deposits in renal tissue from KO mice (Fig. 6H). These observations led to the conclusion that MBD2 deletion alleviates lupus symptoms.

Further analysis focused on whether MBD2 deficiency attenuates lupus symptoms by suppressing humoral immunity in SLE development. The frequencies and numbers of GCB, PBC, PC, and Tfh cells were significantly reduced in KO mice (Fig. 6I–Q). However, other B cell subsets, such as FO, T1 and T2, MZ, and IS B cells, showed no differences (Fig. S4A–G). Additionally, the GC region in KO mice was notably smaller than that in WT mice (Fig. 6R, S). Markers of B cell activation including CD69, CD80, and CD86 were examined and only the proportions of CD69^+^ B cells were lower in KO mice (Fig. 6T–V). Collectively, our findings suggest that MBD2 deficiency mitigates SLE development by inhibiting the GC reaction and humoral immunity-related cell differentiation, thereby reducing autoimmune reactions and subsequent renal injury.

### MBD2 regulates B cell activation and BCR signaling by inhibiting *LEF-1* transcription in SLE

**Fig. 7:**
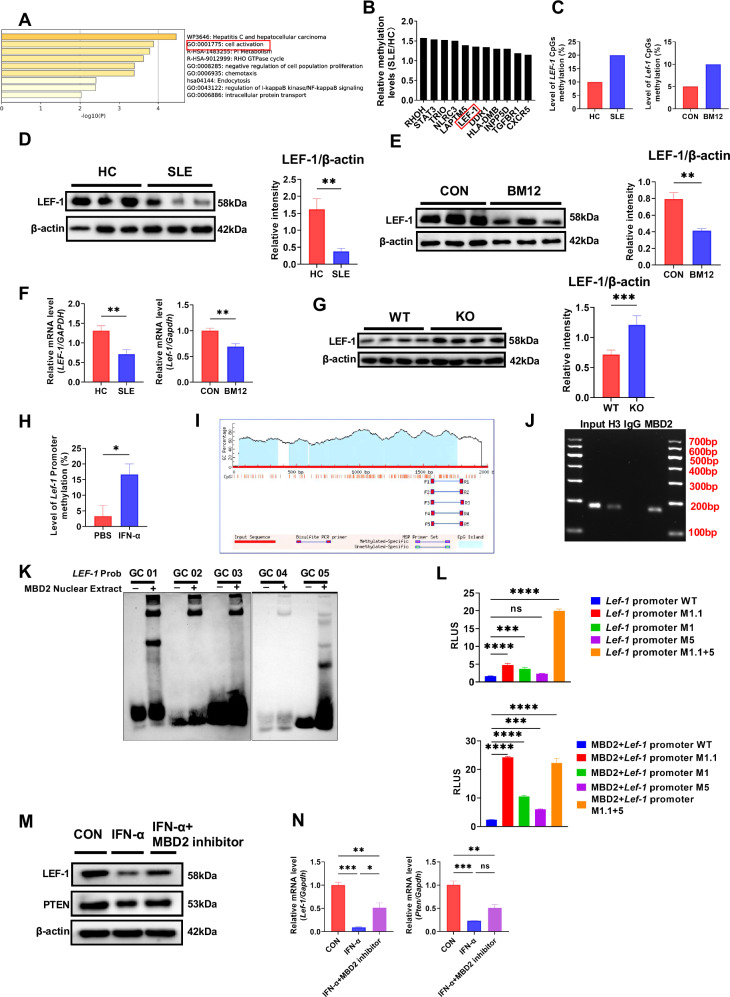
MBD2 regulates B cell activation and BCR signaling by inhibiting *LEF-1* transcription in SLE. **A** GO analysis of 38 genes with increased methylation in SLE B cells from the GSE59250 dataset. **B** Genes with elevated methylation in the cell activation pathway. **C** BSP assay of *LEF-1* (*Lef-1*) CpG methylation in B cells from SLE and lupus model mice. **D** Immunoblot analysis of LEF-1 expression in peripheral blood B cells from SLE patients (*n* = 3) and healthy donors (*n* = 3). **E** Immunoblot analysis of LEF-1 in splenic B cells from Bm12-induced mice (*n* = 3) versus control mice (*n* = 3). **F** RT-qPCR of *LEF-1* (*Lef-1*) mRNA in B cells from SLE patients (*n* = 6), healthy controls (*n* = 6), Bm12-induced lupus models mice (*n* = 3), and control mice (*n* = 3). **G** Immunoblot analysis of LEF-1 in WT and KO mice (*n* = 4). **H** BSP analysis of *Lef-1* promoter CpG methylation dynamics in sorted splenic B cells from WT mice following IFN-α stimulation (50 U/mL, 24 h). **I** Predicted CpG islands of the *Lef-1* promoter using MethPrimer 2.0, with five primer pairs. **J** ChIP demonstrating specific binding of MBD2 protein to the *Lef-1* promoter region in splenic B cells. Input (basic control), H3 (histone H3, positive control), IgG (nonspecific IgG, negative control), MBD2 (specific antibody for target detection). **K** Electrophoretic mobility shift assay of MBD2 binding to *Lef-1*, using biotin-labeled oligonucleotides and nuclear extracts from MBD2-overexpressing HEK293T cells. **L** (Above) Luciferase reporter assay in 293T cells transfected with pGL3 vectors carrying *Lef-1* promoter variants and pRL-TK. (Below) Luciferase assay with additional pCMV-MBD2. Experiments were conducted independently at least three times, with statistical significance determined by two-tailed unpaired Student’s *t-*test. **M** Immunoblot analysis of LEF-1 and PTEN proteins in splenic B cells isolated from WT mice treated with IFN-α (50 U/mL, 24 h), added with or without the MBD2-specific inhibitor KCC07 (10 μM). **N** RT-qPCR analysis of *Lef-1* and *Pten* mRNA levels in sorted splenic B cells from WT mice treated with IFN-α (50 U/mL, 24 h) added with or without the MBD2-specific inhibitor KCC07 (10 μM). *P < 0.05, **P < 0.01, ***P < 0.001, ****P < 0.0001.

MBD2 plays a role in methylation regulation by binding to methylated CpG DNA [8]. However, previous studies have found that there was no methylation of CpG sites within the *PTEN* promoter region in SLE B cells [18], which indicates MBD2 may regulate *PTEN* transcription and expression indirectly. We further re-analyzed the 38 hypermethylated genes from previous research through Gene Ontology (GO) enrichment analysis [18], and found that these hypermethylated genes were primarily concentrated on the cell activation pathway (Fig. 7A) which includes the transcription factor *LEF-1* (Fig. 7B). Additionally, our previous research has found that LEF-1 interacts with WASp to inhibit its phosphorylation, thereby affecting B cell activation and BCR signaling [31]. To verify whether MBD2 regulated B cell activation and BCR signaling through binding to methylated CpG sites within the *LEF-1* promoter, we conducted BSP sequencing and verified the methylation levels of CpG sites in the promoter regions of *LEF-1*. Results confirmed elevated methylation levels at CpG sites in the *LEF-1* promoter regions in B cells from SLE patients and lupus model mice compared to healthy individuals and normal mice (Fig. 7C). And the expression of LEF-1 is significantly reduced in B cells from both SLE patients and cGVHD model mice (Fig. 7D, E). The RT-qPCR result also showed mRNA of *LEF-1* was downregulated (Fig. 7F). Furthermore, immunoblotting indicated an upward trend in LEF-1 protein levels in KO B cells compared to WT B cells (Fig. 7G). These results suggest that MBD2 may promote B cell activation and BCR signaling by inhibiting *LEF-1* transcription in SLE.

IFN-α was reported to participate in the DNA methylation regulation [7]. IFN-α is significantly increased in SLE patients, which influences the onset and progression of SLE by promoting B-cell survival, proliferation, and differentiation, strongly correlated with SLE disease activity [33]. To verify whether the elevated levels of IFN-α in lupus contribute to the hypermethylation of *Lef-1*, we stimulated mouse splenic B cells with IFN-α for 24 h, and found increased methylation levels at the CpG site in the *Lef-1* promoter region (Fig. 7H). We further investigated whether MBD2 regulates *Lef-1* transcription by binding to its methylated CpG sites. MethPrimer 2.0 analysis identified multiple CpG islands in the *Lef-1* promoter sequence, and we designed several primer pairs corresponding to different regions (Fig. 7I). MBD2 could bind to the *Lef-1* promoter region in B cells stimulated with IFN-α (Fig. 7J). Methylated CG sites in the *LEF-1* promoter region were used to predict binding sites (Fig. S5A). Electrophoretic mobility shift analysis (EMSA) further revealed that MBD2 bound to regions 1 and 5 of the *Lef-1* promoter (Fig. 7K). Using the binding site as a reference, we designed multiple mutation sites for validation (Fig. S5B, C). DNA methylation-dependent luciferase reporter gene analysis confirmed that *Lef-1* transcriptional activity was higher in cells transfected with the mutant plasmid (Fig. 7L). Lastly, the expression and transcription levels of LEF-1 in B cells following IFN-α stimulation were reduced, while it was restored with the intervention with the MBD2 inhibitor KCC07 (Fig. 7M, N). These findings suggest that MBD2 inhibits *Lef-1* transcription and expression following IFN-α stimulation.

### MBD2 regulates PTEN expression by inhibiting *Lef-1* transcription

**Fig. 8:**
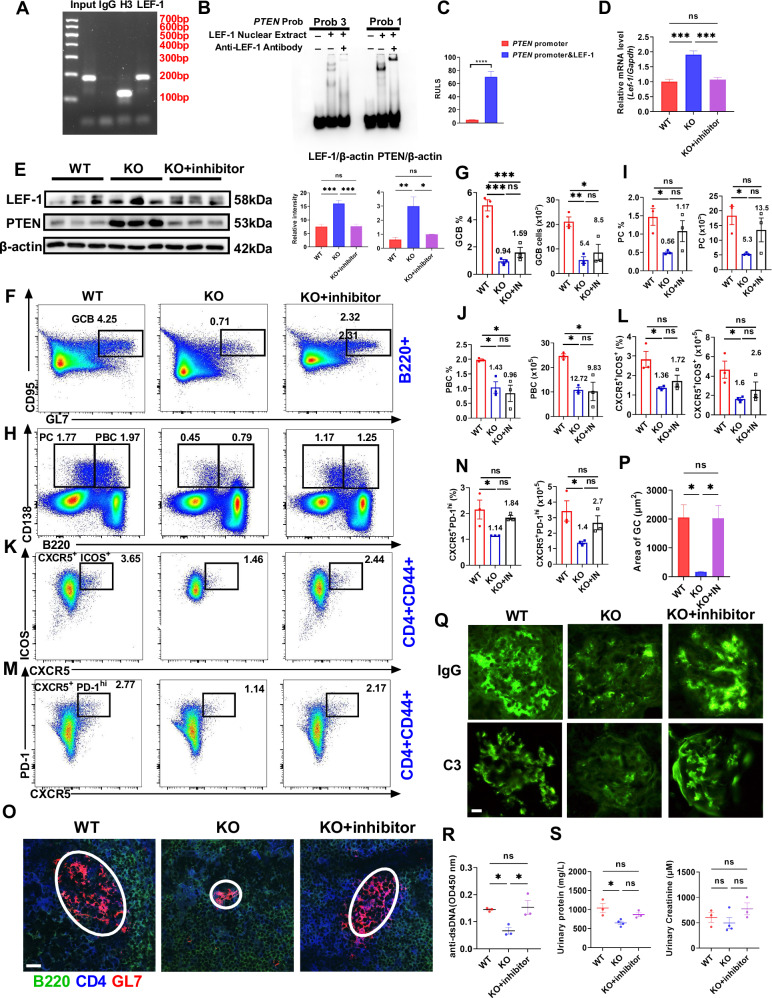
MBD2 regulates PTEN expression by inhibiting *Lef-1* transcription. **A** ChIP analysis of LEF-1 binding to the *Pten* promoter. Input (basic control), H3 (histone H3, positive control), IgG (nonspecific IgG, negative control), LEF-1 (specific antibody for target detection). **B** Electrophoretic mobility shift assay of LEF-1 binding to *Pten*. **C** Luciferase reporter assay in 293T cells transfected with pGL3 vectors carrying *Pten* promoter variants and pRL-TK with or without LEF-1. **D** RT-qPCR of *Lef-1* and *Pten* expression in splenic B cells from WT (*n* = 3), KO (*n* = 3), and KO + LEF-1 inhibitor mice (*n* = 3). **E** Immunoblot analysis of LEF-1 and PTEN expression in splenic B cells from WT (*n* = 3), KO (*n* = 3), and KO + LEF-1 inhibitor mice (*n* = 3). **F**–**N** Representative flow diagrams and statistical analysis of GC B cells, PBC, PC, Tfh, B cells in spleens from Bm12-induced WT (*n* = 3), KO (*n* = 3), and KO + LEF-1 inhibitor mice (*n* = 3). **O**, **P** Immunofluorescence of GCs in spleens from Bm12-induced WT, KO, and KO + LEF-1 inhibitor mice (*n* = 3), with anti-CD4 (blue), anti-B220 (green), and anti-GL-7 (red) (scale bar, 20 μm). **Q** Immunofluorescence of C3 and IgG in kidneys from Bm12-induced WT mice, KO mice, and KO + LEF-1 inhibitor mice (*n* = 3, scale bar, 10 μm). **R** ELISA analysis for anti-dsDNA antibody levels in serum from Bm12-induced WT (*n* = 3), KO (*n* = 3), and KO + LEF-1 inhibitor mice (*n* = 3). **S** Urinary protein and creatinine analysis in Bm12-induced WT (*n* = 3), KO (*n* = 3), and KO + LEF-1 inhibitor mice (*n* = 3). *P < 0.05, **P < 0.01, ***P < 0.001, ****P < 0.0001.

To further verify whether LEF-1 regulates *Pten* transcription and expression, ChIP assays were performed to explore the relationship between LEF-1 and *Pten*, the result confirmed that LEF-1 could bind to the *Pten* promoter region (Fig. 8A), and EMSA assay similarly showed the binding relationship between LEF-1 and *Pten* (Fig. 8B). Besides, the transcriptional activity of *Pten* was higher in transfected cells containing the LEF-1-overexpression plasmid than in cells without the LEF-1-overexpression plasmid (Fig. 8C). Furthermore, IFN-α similarly repressed the *Pten* transcription and expression (Fig. 7M and N). These findings indicate that LEF-1 promotes *Pten* transcriptional regulation and expression, suggesting a potential role in SLE pathogenesis.

Next, we observed a significant reduction in the transcription and expression levels of LEF-1 and PTEN in the KO splenic B cells treated with LEF-1 inhibitor when compared to untreated KO splenic B cells (Fig. 8D, E). Additionally, the proportions of GCB, PC, PBC, and Tfh cells were recovered to some extent in inhibitor-treated KO mice compared with untreated KO mice, although without significant differences (Fig. 8F–N). Compared to the KO group, treatment with inhibitor exhibited an enlarged area of GC in the spleens, increased deposition of complement C3 and IgG in kidney tissues, and noticeably enlarged spleens (Fig. 8O–Q and Fig. S6A). Levels of anti-dsDNA antibodies and various kidney injury markers were upregulated, suggesting that the LEF-1 inhibitor effectively aggravates lupus symptoms in KO mice (Fig. 8R, S). Kidney tissues revealed increased cellular infiltration in the glomeruli of KO mice treated with the inhibitor (Fig. S6B). These findings above indicate that the MBD2 regulates PTEN expression by inhibiting *Lef-1* transcription in lupus.

## Discussion

In our study, MBD2 expression was elevated in B cells from SLE patients and positively correlated with GC B cells and PBC cells linked to SLE pathogenesis. MBD2 regulates B cell activation and BCR signaling via the LEF-1-PTEN axis. In KO mice, reduced GC B cells and plasma cells were observed, along with decreased antibody secretion during humoral immunity and alleviated lupus symptoms in the cGVHD model. These findings underscore the critical role of MBD2 in SLE pathogenesis.

Multiple studies have established a strong link between DNA demethylation and SLE onset. A genome-wide DNA methylation analysis of 154 SLE patients revealed significant demethylation specifically in CD4^+^ T cells, unlike in CD8^+^ T cells and peripheral monocytes [34, 35]. IFN-α levels were elevated in SLE patients, and promoter methylation of the interferon-regulated gene IFI44L was identified as a sensitive and specific biomarker for SLE diagnosis [36]. Aberrant methylation was less frequent in B cells than in T cells. Detailed analysis revealed that MBD2 binds to the methylated CpG region of the *Lef-1* promoter, suppressing *Lef-1* and *Pten* transcription and translation, thereby affecting B cell development and differentiation. These findings provide new insights into the molecular mechanisms of SLE and highlight the importance of further exploring B cells in SLE research.

The results of this study demonstrate that MBD2 deficiency affects the differentiation of GC B cells in the GC. This is due to the fact that naïve B cells are the starting point for B cell differentiation in peripheral organs. However, upon analysis of the proportion of naïve B cells and the number of B cells in WT and KO mice, no significant differences were observed in the percentage of naïve B cells or the expression levels of IgM and IgD in naïve B cells between the two groups (data not shown). This finding indicates that the deletion of MBD2 in naïve B cells is not the causative factor for the abnormalities in B cell differentiation.

Abnormal BCR signaling is pivotal in the pathogenesis of SLE [37]. The PI3K pathway, critical for B-cell development and activation, orchestrates various downstream signaling molecules and is primarily regulated by the phosphatases PTEN and SHIP-1 [28]. Increased PTEN expression was detected in MBD2 knockout mice, which in turn reduced PI3K and downstream Akt expression. Notably, prior research has shown that MBD2 modulates the PI3K signaling pathway in macrophages through SHIP repression in lung fibrosis [38]. The MBD2 gene deletion impairs PI3K/Akt pathway activation in breast cancer. Additionally, the PI3K/Akt pathway becomes abnormally activated in response to IFN-α [39, 40]. In the cGVHD model, we found that MBD2 modulates the PI3K pathway through PTEN. Indeed, to validate whether PTEN loss can rescue the MBD2 phenotypes, a PTEN inhibitor was used for in *vitro* inhibition experiments. We found that decreased BCR signaling in KO B cells were restored after incubated with PTEN inhibitor, which was consistent with our previous findings. This suggests that the regulation of the PI3K pathway by MBD2 may differ across diseases and immune cell types. These results highlight the crucial role of MBD2 in B-cell function and SLE pathogenesis.

Previous studies have highlighted the role of MBD2 in suppressing PBCs differentiation in vitro [41], yet its impact on PBCs remained unverified in vivo. The PBC/PC ratio significantly decreased in MBD2 knockout mice in NP-KLH and cGVHD models, confirming the involvement of MBD2 in PBC differentiation in vivo. Increased MBD2 expression in PBCs was observed in SLE patients, with a positive correlation to SLEDAI. B-cell hyperactivation plays a crucial role in SLE pathogenesis. Previous research demonstrated that LEF-1 inhibits B-cell activation and signaling by binding to WASp and blocking its phosphorylation [31].

Our study revealed that LEF-1 in an SLE model exhibited increased methylation, reduced transcript levels, and impaired actin reorganization and BCR signaling via PTEN enhancement. Numerous studies highlight the critical role of IFN-α-induced demethylation in disease onset and progression. We confirmed that IFN-α-induced hypermethylation in lupus significantly inhibits B cell activation and signaling, which is key to disease development [42]. LEF-1 inhibitors in MBD2 knockout mice induced significant lupus-like symptoms, highlighting the critical role of LEF-1 in SLE pathogenesis. One limitation of this study is that treatment with the LEF-1 inhibitor triggered unexpected side effects during the development of the lupus model, leading to higher mortality in the treatment group and undermining the statistical differences between groups, the other limitation of this study is the absence of a *Lef-1*^fl/fl^ *Cd19*^cre/+^ mouse model for more detailed research. Future studies should consider using primary models such as the MRL/lpr mouse for broader investigation, although the cGVHD model confirmed the connection between MBD2 and LEF-1.

Previous research identified elevated MBD2 expression in B cells associated with lupus nephritis [43–45]. This study further discovered that in lupus patients experiencing nephritis, expression levels of MBD2 in DN B cells, PBC cells, and general B cells were markedly higher compared to those in patients without nephritis. These results highlight the potentially crucial role of MBD2 in lupus nephritis, although the exact mechanisms require additional investigation. The study limitations, including a small sample size and insufficient differentiation among various demographic groups (children, women, and men), might limit the generalizability of the results. However, this research contributes valuable insights into the involvement of MBD2 in lupus and underscores the potential of B-cell targeted therapies in treating lupus patients.

## Supplementary information

Supplementary figure legend

Supplementary Figure1

Supplementary Figure2

Supplementary Figure3

Supplementary Figure4

Supplementary Figure5

Supplementary Figure6

Supplementary Figure7

Supplementary Figure8

Supplementary Figure9

Supplementary Table

## Data Availability

All data generated or analyzed during the course of this study are included in the published article information file, as well as in the supplementary information file.
