# Supplementary material for: MBD2 promotes B cell differentiation and BCR signaling in systemic lupus erythematosus by regulating the LEF-1-PTEN-PI3K axis
Source: Cell Death Dis. 2025 Jun 4;16(1):433. doi: 10.1038/s41419-025-07750-6 (PMC12137598; doi:10.1038/s41419-025-07750-6)
Supplement: Supplementary file 1 — Supplementary figure legend [file 41419_2025_7750_MOESM1_ESM.docx]

**Supplementary legend**

**FigS1. Gating strategies for human peripheral blood B cell subsets and mouse bone marrow B cell subsets.**

A. The Gating strategy for B-cell subsets in PBMCs from SLE patients and healthy controls. B. The Gating strategy for bone marrow B cell subsets in WT and KO mice.

**FigS2. Splenic B-cell apoptosis and proliferation in WT and KO mice and PTEN inhibitor effects on KO B cells.**

A. Representative flow cytometry diagrams showing apoptotic (Annexin V) and proliferative (Ki-67) in total, FO, IS, MZ, T1, and T2 B cells from the spleens of WT and KO mice. B. Immunoblot analysis of BCR signaling molecules in WT, KO, and KO+ PTEN inhibitor B cells.

**FigS3. Deficiency in MBD2 attenuates the T-cell-dependent immune response.**

A-H. Representative flow diagrams and statistical analysis of FO, T1, T2, IS, and MZ B cells in spleens of immunized WT (n=6) and KO mice (n=6).

**FigS4. MBD2 deficiency ameliorates lupus-like autoimmunity and renal injury in a cGVHD model.**

A-G. Representative flowcharts and statistical analysis of FO, T1, T2, IS, and MZ B cells in spleens from Bm12-induced WT (n=6) and KO mice (n=6). H. Spleen weight/Total weight index in WT (n=6) and KO mice (n=6) in the cGVHD lupus model.

**FigS5. LEF1 promoter site prediction and mutant design in the lef-1 promoter region.**

A. Binding region (from -1529bp to +1bp, with a transcription start site of +1) of methylated CpG DNA was found in the *Lef-1* promoter. B. Methylated CG sites in the LEF1 promoter. C. Mutation sites in the LEF1 promoter.

**FigS6. MBD2 regulates PTEN expression by inhibiting Lef-1 transcription.**

A. Spleen images from Bm12-induced WT mice, KO mice, and KO + LEF-1 inhibitor mice. B. HE staining of kidneys from Bm12-induced WT mice, KO mice, and KO + LEF-1 inhibitor mice (scale bar, 60 μm).

**FigS7-S9.** The raw data for the Western blot (WB), electrophoretic mobility shift assay (EMSA), and chromatin immunoprecipitation (ChIP) experiments are provided in the article.
