# Supplementary figures and images for: MBD2 promotes B cell differentiation and BCR signaling in systemic lupus erythematosus by regulating the LEF-1-PTEN-PI3K axis
Source: Cell Death Dis. 2025 Jun 4;16(1):433. doi: 10.1038/s41419-025-07750-6 (PMC12137598; doi:10.1038/s41419-025-07750-6)

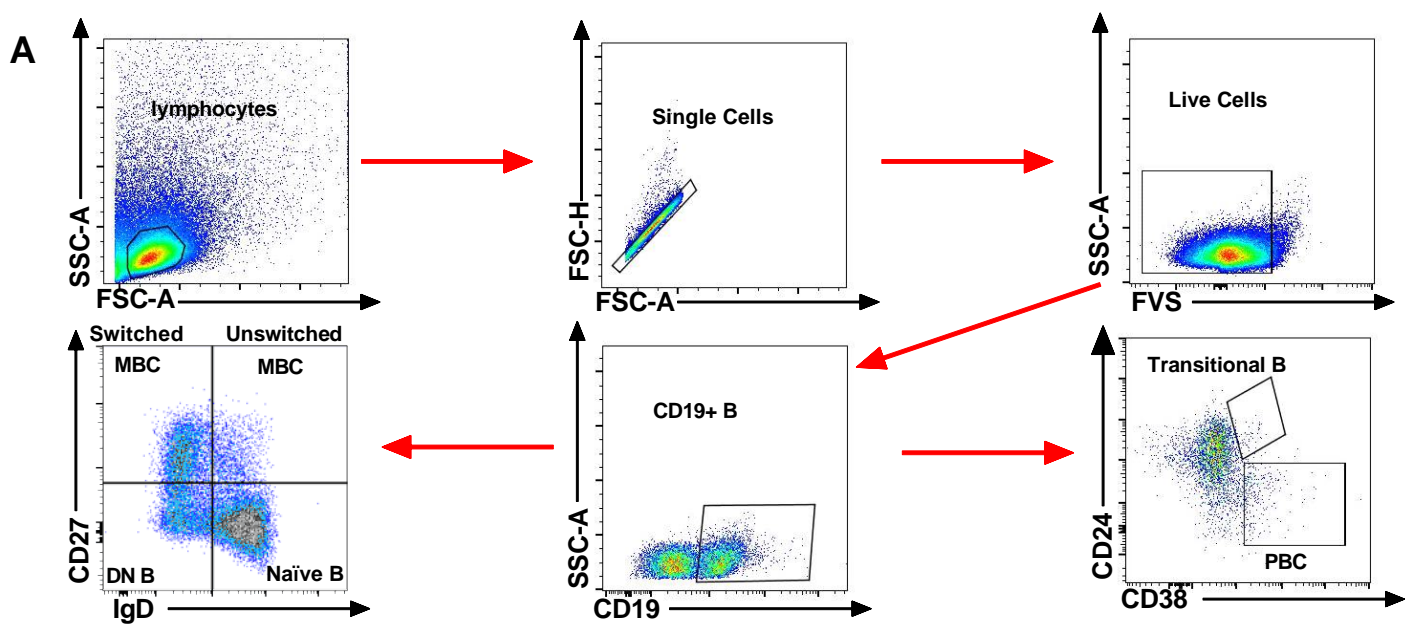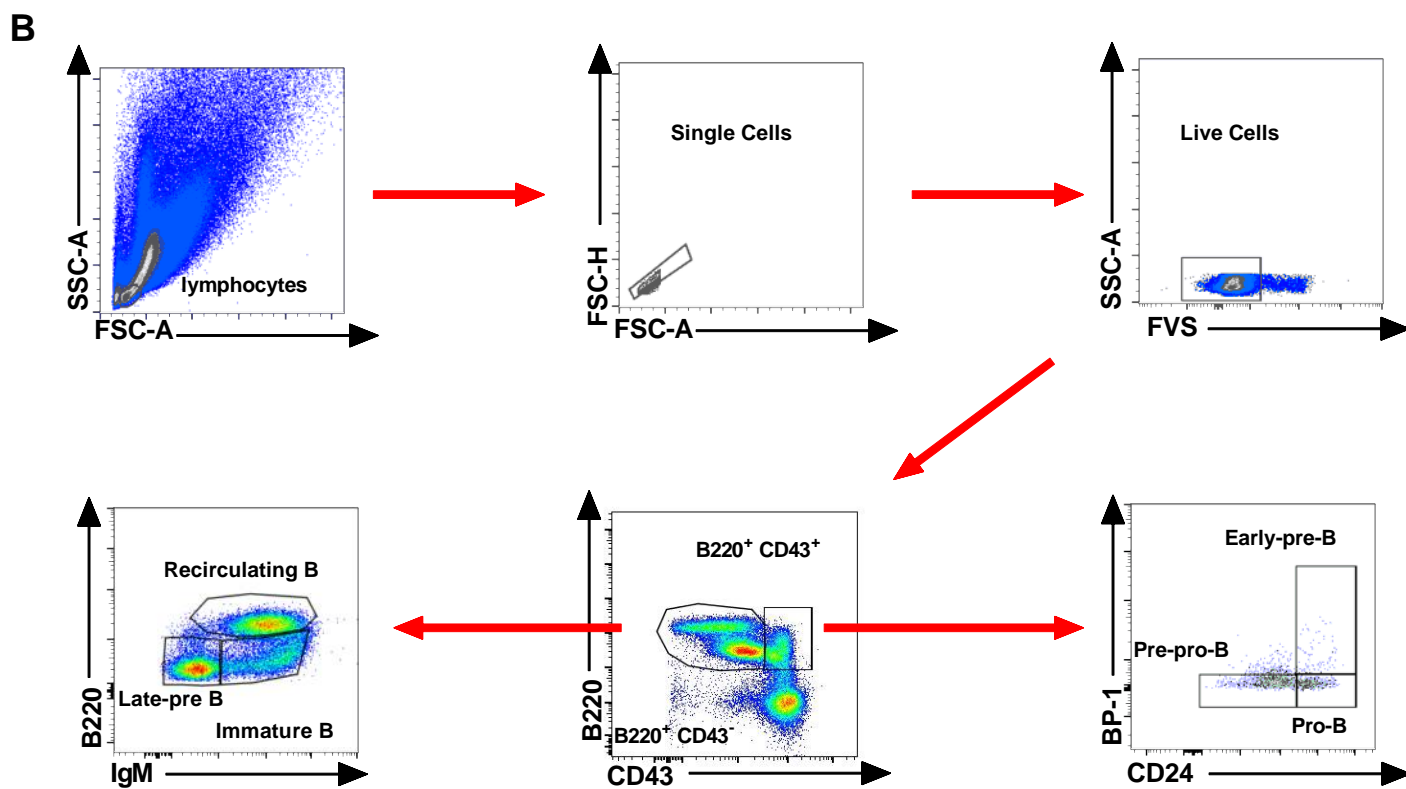

Supplement: Supplementary file 2 — Supplementary Figure1 [file 41419_2025_7750_MOESM2_ESM.pdf]

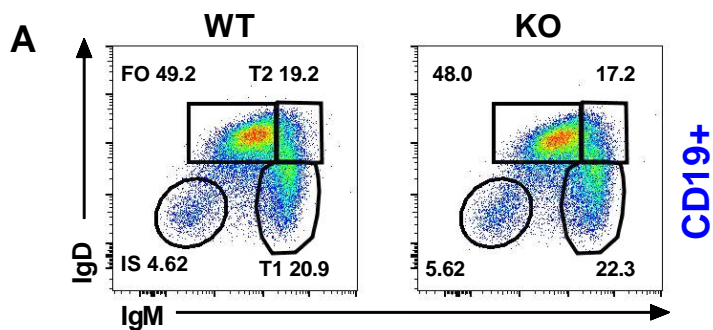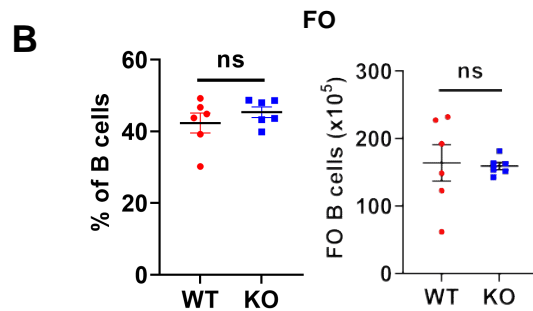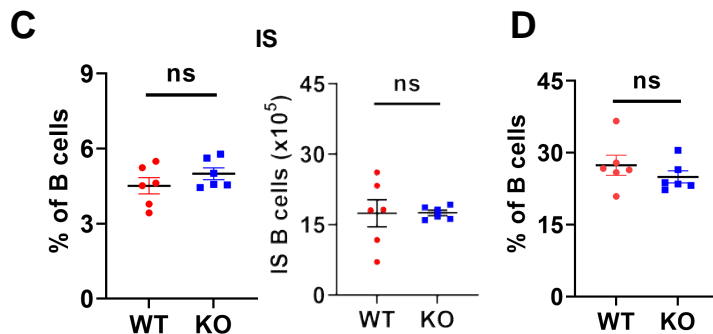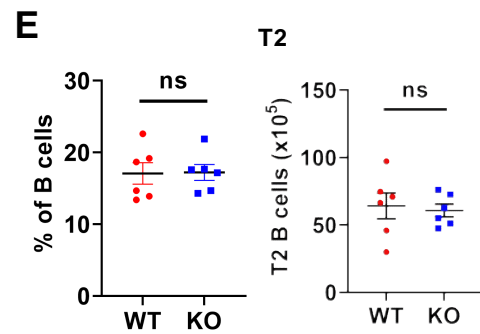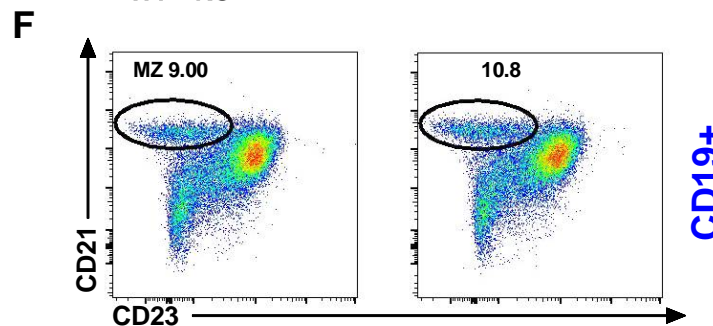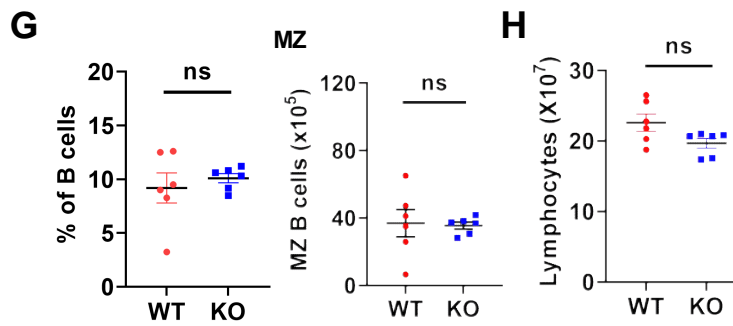

Supplement: Supplementary file 4 — Supplementary Figure3 [file 41419_2025_7750_MOESM4_ESM.pdf]

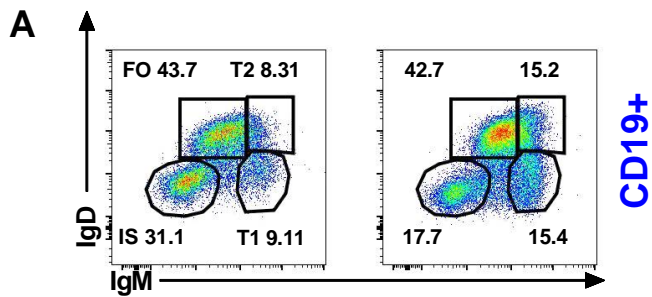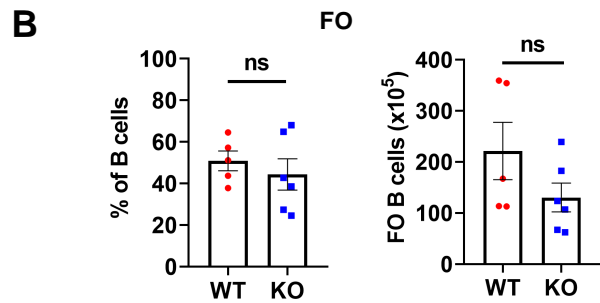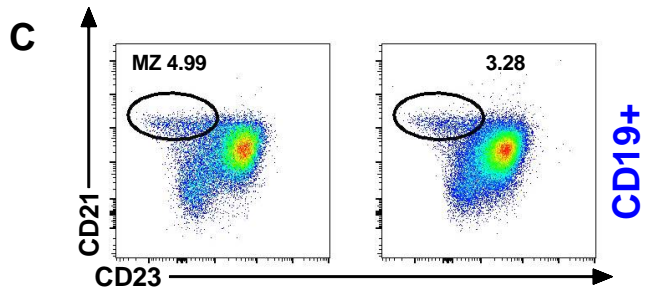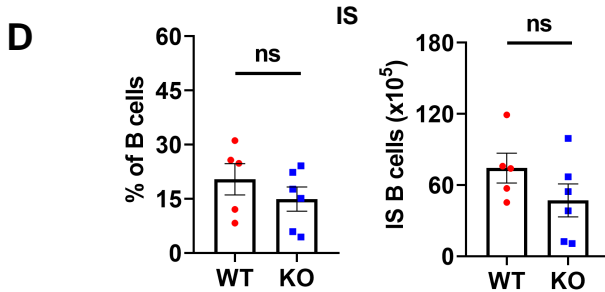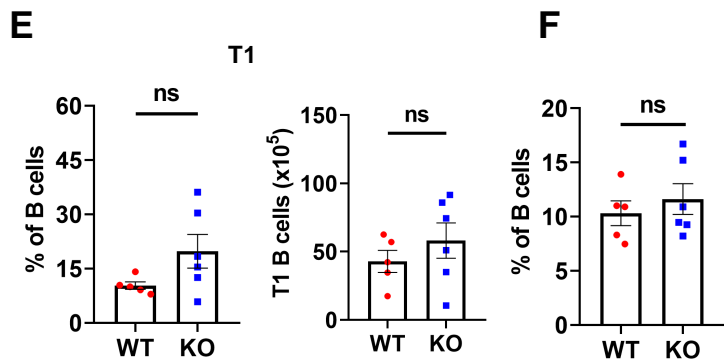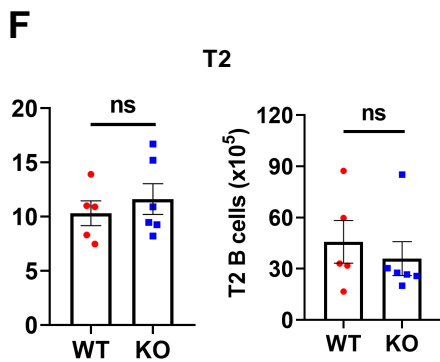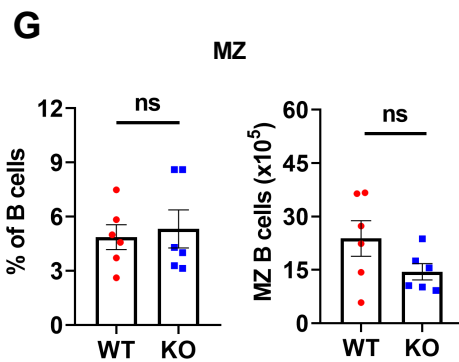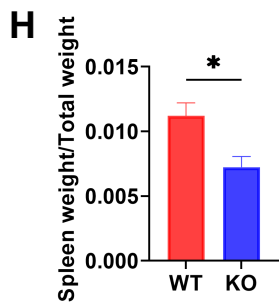

Supplement: Supplementary file 5 — Supplementary Figure4 [file 41419_2025_7750_MOESM5_ESM.pdf]

**A**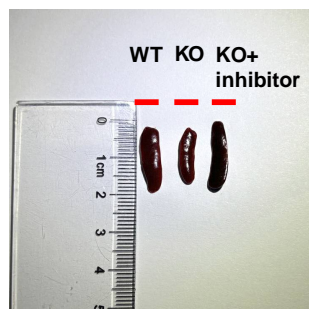**B**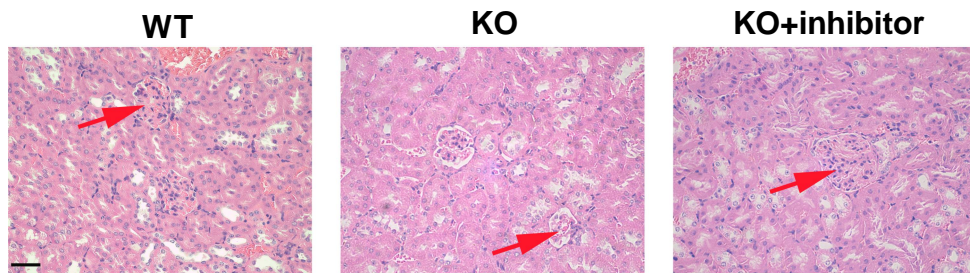

Supplement: Supplementary file 7 — Supplementary Figure6 [file 41419_2025_7750_MOESM7_ESM.pdf]

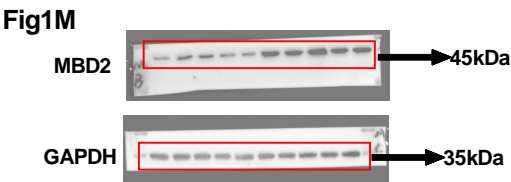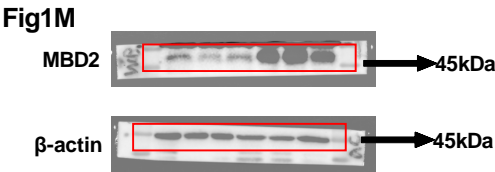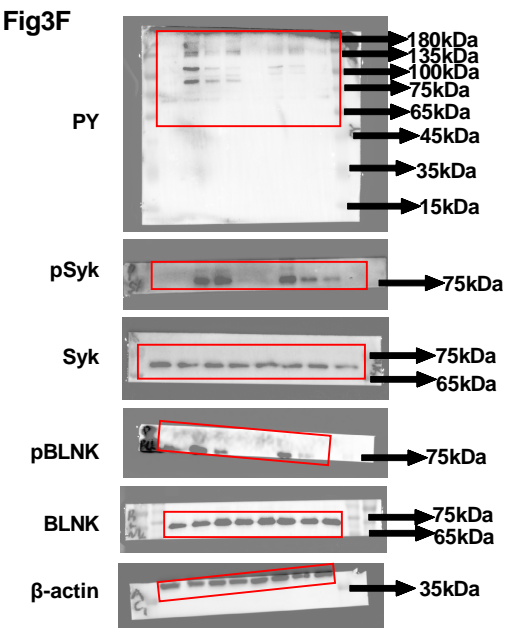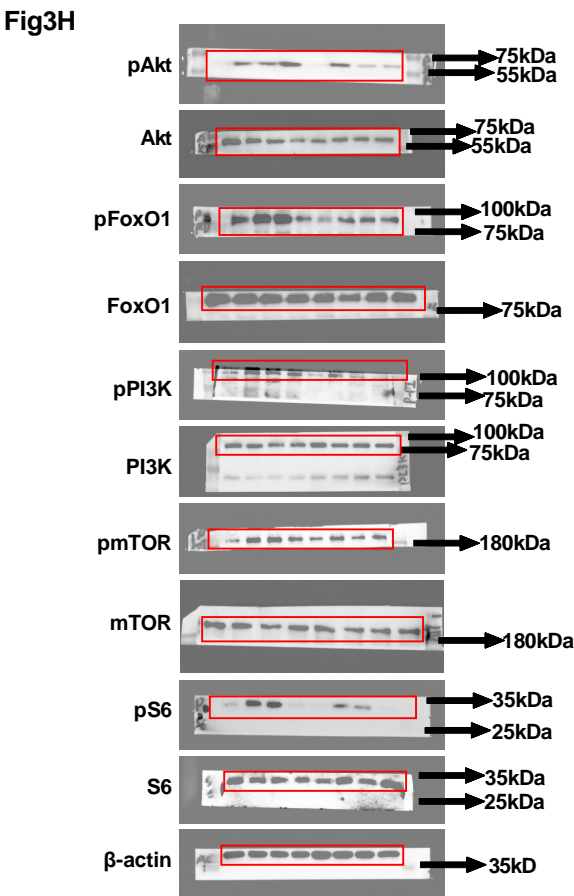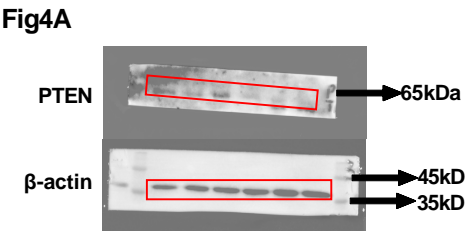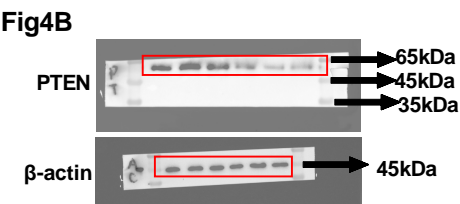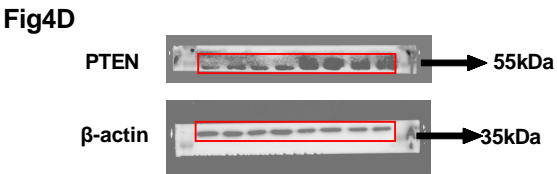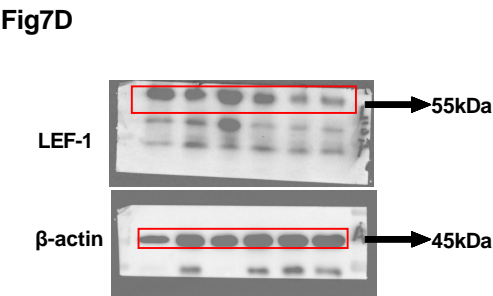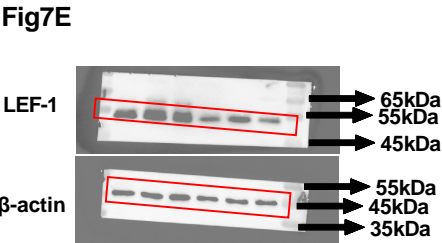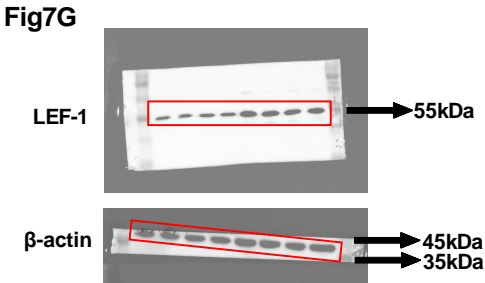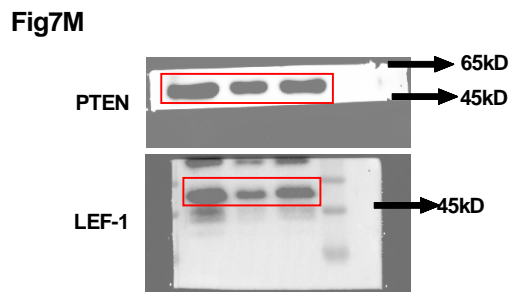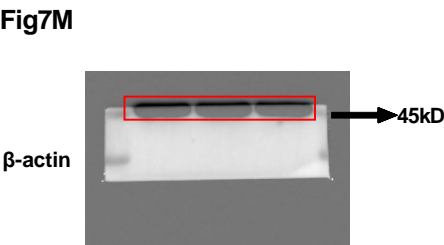

Supplement: Supplementary file 8 — Supplementary Figure7 [file 41419_2025_7750_MOESM8_ESM.pdf]

Fig7J

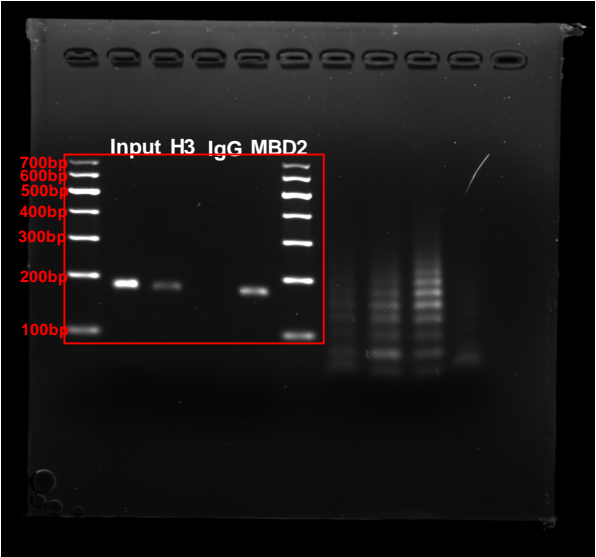

Fig7K

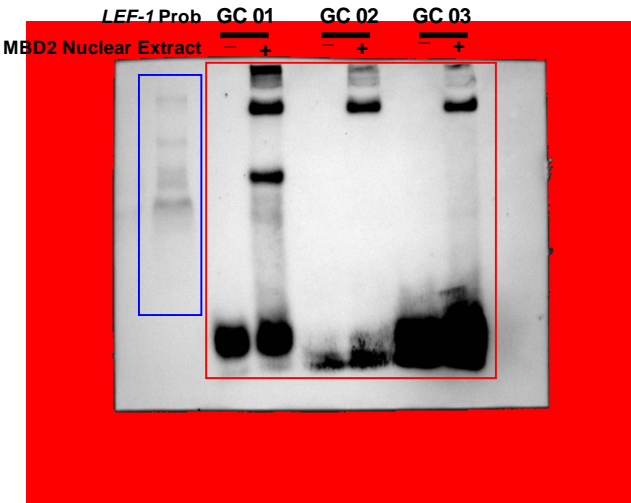

Fig7L

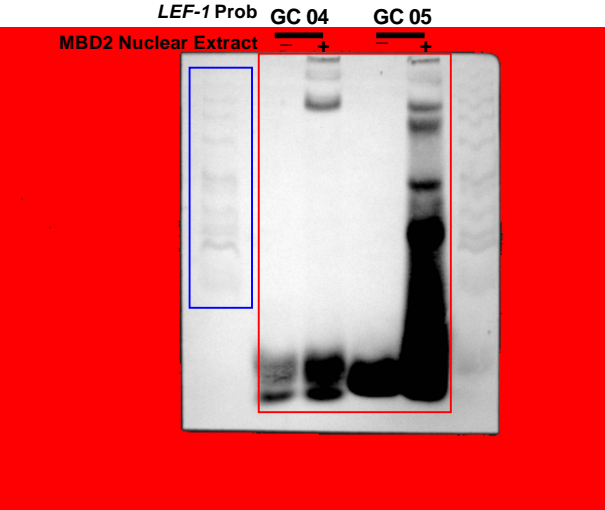

Fig8A

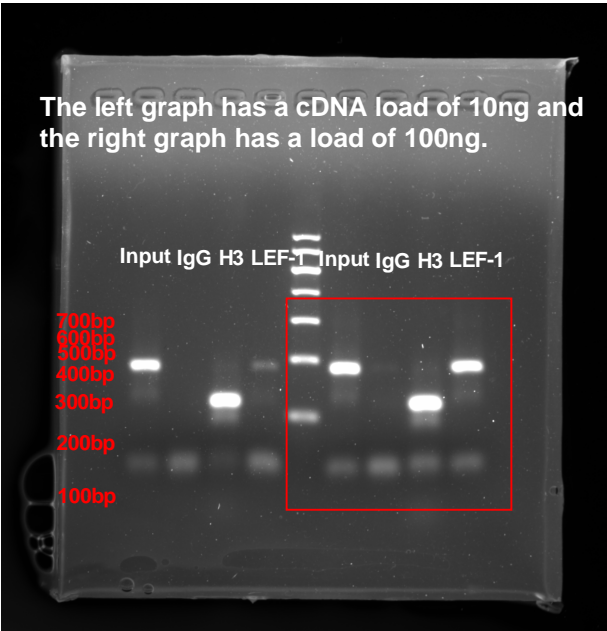

Fig8B

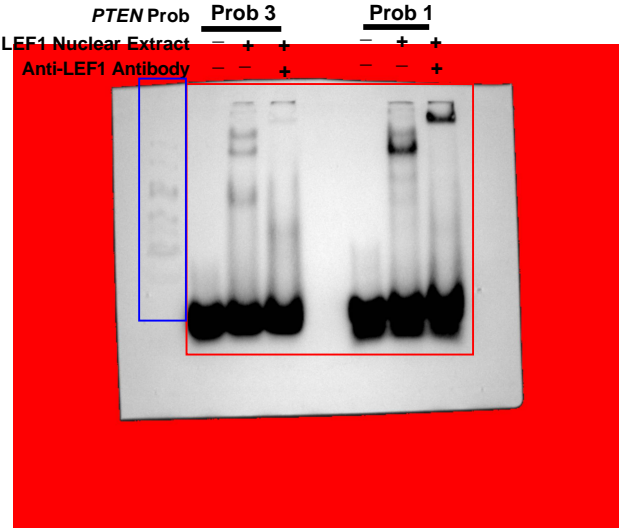

Fig8E

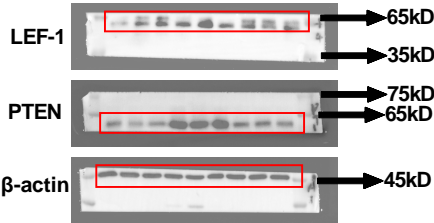

Supplement: Supplementary file 9 — Supplementary Figure8 [file 41419_2025_7750_MOESM9_ESM.pdf]

**FigS2B**

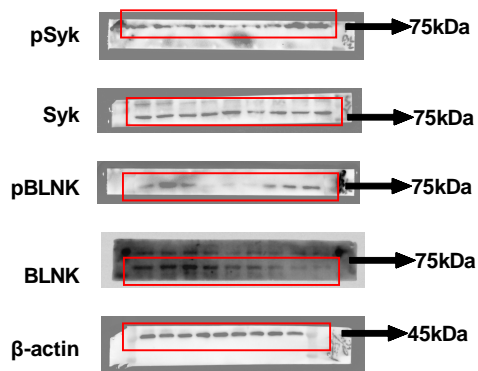

**FigS2B**

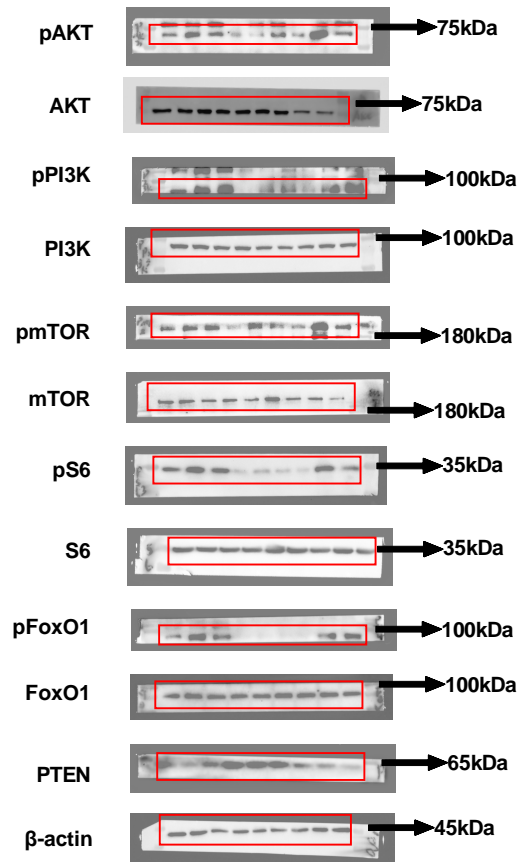

Supplement: Supplementary file 10 — Supplementary Figure9 [file 41419_2025_7750_MOESM10_ESM.pdf]
