# Supplementary material for: MBD2 promotes B cell differentiation and BCR signaling in systemic lupus erythematosus by regulating the LEF-1-PTEN-PI3K axis
Source: Cell Death Dis. 2025 Jun 4;16(1):433. doi: 10.1038/s41419-025-07750-6 (PMC12137598; doi:10.1038/s41419-025-07750-6)
Supplement: Supplementary file 6 — Supplementary Figure5 [file 41419_2025_7750_MOESM6_ESM.pdf]

A

GCCATTCCCGTAGTTTCTCGTCAATTTTCACGTGGGCCACACTGTTCTTTGGGGTTGGGTTACACACAGCAC
CCAGTCTATCTTCAGGTTGTATGGTGTCGTCCCCCCCCCCCCAGGCATGAACAGGCGGGCGCAAACACATAC
GCACCCCCACGGCGCCCCCCCCCCCCAAGTATGACTCGTCCTTTGGGTGCTAGGAAATGAACTAGGGCTGG
GGGGGGGGGGAGAGAACCTGGATCTCCTCCTCCTCCTAACAGACTCCCTCCTCCCCACCCCTCCAGCAGGC
AGCCAAGGAGAGCAGGAGGCGGGGGAGGGGCGGGGGAGGAGAAGCGACGCAAGTGAATAGCTTTGCA
GCGCCGGCTAGGCGCGGCGAGGAGAAGCGGTGGGAAGGCGCAACCGCTCACCTGCGGGGCAGCGCGCA
CCGGAGGGACCGGGCTGCGCTTTCGTGGGCCCTGGCATTCGGACATTCCCGGAGCCTTGCAAGCGGCTAC
GCTCCGGGTGTTCCCCGGCCGGTCTGCCCGGAGGAGCCGAGGGGCCACCGGCAGCTCTTTGCTTTGACCG
AGGGGAGGCGGGGAGAGAGGCGAGCCAGGCAGAAACTCGAGCAACTCGAGC**<u>CGGGAACAAAGAGGGA</u>**
<u>TCGG</u>GCTCGAGTGTGTGTGTGTGTGTGTGTGTGTGTGTGTGTGTGTAAGTGTGTGTATCGGCCCGAGCTGCG
GGCTGGAACATTTGGGCCCGAAGCTCCTGCTGTGACTCCCCAAGACTCCGCCGTGCCAGCCAC**<u>CGCCGATT</u>**
<u>CCCAGCGCTCATCATCA</u>CAAACTTTATTCTTGGCAAACTTCTCTTTTTCTCCCCTCCTCCTCCGCCCCTCCCCCT
CCAGCAGATTAAATGCTCCTCCAGAAGGAAAACCGAAGCGAAAGGGAAGGAAAGAAGCTCTAACGCGGA
CGTCTGCAGCCCGGTGGCTCTTTATTGTTTACTCTGAAGGAAGTGGACTTTTCGGTATTTTCTGATTCTTCTC
GTACCTCCGCTGGGGCAAAGGGAGCCTCTTGGCCCAGCTCTCCTCTTCTCAAAAACAAAACAAAACAAAAC
CCCAAATCACCGAAATCAGCATCCGAGGAATCCGTGGAGAGGTTTTGCACGGTGACGCACCACGGGACCCT
CGTTTGCACTTTGGCAAGTCGTGTCCCCTCCGGAACCGGTGTCCTCGAAGCACAGACCCCAGCAACGAGTG
CGGCGTGGGGTGGCCACCCAGCCCTGGCAGCCTAGCCTAGTGCACGCGGAGCGGCGTAGACGCTCTCAGA
GGCGCCGGCGGCTGCCGCCCTCGCAGTCTGGTGCCCTTCGCCCCAGGGCCGGGCGCCGGCGTCCTTCTAA
GTGGGAAAGCGCGGCAATCGCAGAGGCTCCTGCAGCTGCTCCAGCCGTGGGCCCCTCGCTTGCCCACCCG
GCTTCCATTCACAGTCCTCCCGCACTCAGTCTTCCAACTCCCCCACCTCACATCGACCCTCCCTTCTTGCTGTC
TCGGAGTGCTCCCGCAGCGGAGCGGAGATTACACAGCCGCCGGG**ATG**

+1

Yellow areas are CHIP binding sites

<u>Underlined areas are EMSA positive region 1 and positive region 2</u>

Red arrows indicate methylation and mutation sites

Blue areas are other predicted binding sites of the CHIP assay

B

***Lef-1 GC01-01*-1.1**	**<u>CGCCGATTCCCAGCGCTCATCATCA</u>**
***Lef-1 GC01*-1**	**<u>CGGGAACAAAGAGGGATCGG</u>**
***Lef-1 GC05*-5**	**<u>CGCCGATTCCCAGCGCTCATCATCA</u>**

C

***Lef-1 promter* M1.1**	**<u>AGCCGATTCCCAGCGCTCATCATCA</u>**
***Lef-1 promter* M1**	**<u>AGGGAACAAAGAGGGATAGG</u>**
***Lef-1 promter* M5**	**<u>AGCAGATTCCCAGAGCTCATCATCA</u>**
