# Supplementary material for: MBD2 promotes B cell differentiation and BCR signaling in systemic lupus erythematosus by regulating the LEF-1-PTEN-PI3K axis
Source: Cell Death Dis. 2025 Jun 4;16(1):433. doi: 10.1038/s41419-025-07750-6 (PMC12137598; doi:10.1038/s41419-025-07750-6)
Supplement: Supplementary file 11 — Supplementary Table [file 41419_2025_7750_MOESM11_ESM.docx]

**Table S1: Primer sequence of *Lef1* mutants**

| Name | Sequence |
| --- | --- |
| Lef1-m1.1-F1 | GATCTAAACTCGAGCAGGGAACAAAGAGGGA |
| Lef1-m1.1-F2 | TCCCTCTTTGTTCCCTGCTCGAGTTTAGATC |
| lef1-mut1-F | GATCTAAACTCGAGCaGGGAACAAAGAGGGATaGGGCTaGAGTGTGTGTGTGTGT |
| lef1-mut1-R | ACACACACACACACTCtAGCCCtATCCCTCTTTGTTCCCtGCTCGAGTTTAGATC |
| lef1-mut5-F | CGCCGTGCCAGCCACaGCaGATTCCCAGaGCTCATCATCACAAAC |
| lef1-mut5-R | GTTTGTGATGATGAGCtCTGGGAATCtGCtGTGGCTGGCACGGCG |

**Table S2:Primers sequence for PCR, RT-qPCR and CHIP-qPCR**

| Primer | Sequence 5’-3’ | BLAST RID |
| --- | --- | --- |
| *RT-H-PTEN-F*  *RT-H-PTEN-R* | TGTGGTCTGCCAGCTAAAGG  CGGCTGAGGGAACTCAAAGT | 2H8PZDKS013  2H8PZDKS013 |
| *RT-H-LEF1-F*  *RT-H-LEF1-R* | GCCTACATCTGAAACATGGTGG  GCGTCTCTAGCAGTGACCTC | 2H9E06CD013  2H9E06CD013 |
| *H-MBD2-F*  *H-MBD2-R* | CCATGGAACTACCCAAAGGTCTT  CAGCAGATAAAAGGGTCTCATCATT | 2H9NJANS01R  2H9NJANS01R |
| *GAPDH* RT-qPCR(F) | GCAAATTCCATGGCACCGT | KVYF4KGA016 |
| *GAPDH* RT-qPCR(R) | GCCCCACTTGATTTTGGAGG | KVYF4KGA016 |
| *RT-M-LEF1-F*  *RT-M-LEF1-R*  *RT-M-PTEN-F*  *RT-M-PTEN-R* | TGAGTGCACGCTAAAGGAGA  CTGACCAGCCTGGATAAAGC  GCAGCTTCTGCCATCTCTCTC  AACGATCTCTTTGATGATGGCTG | 2HHA8FDB013  2HHA8FDB013  2HB20J3C01R  2HB20J3C01R |
| *M-MBD2-F*  *M-MBD2-R* | GGAGGAAGTGATCCGAAAATCAG  AGCATTTCCCAGGTATCTTGC | 2HB2HA3P01R  2HB2HA3P01R |
| *Gapdh* RT-qPCR(F)  *Gapdh* RT-qPCR(R) | GGTGAAGGTCGGTGTGAACG  CTCGCTCCTGGAAGATGGTG | KW117WD4016  KW117WD4016 |
| CHIP-MBD2 *Lef-1-*promter-F2-F | CGGGAACAAAGAGGGATCGG |  |
| CHIP-MBD2 *Lef-1-*promter-F2-R | TGATGATGAGCGCTGGGAAT |  |
| CHIP-LEF-1*-pten-* promter F1 | TCTGCCGGGTTTTCATAGCG |  |
| CHIP-LEF-1*-pten-* promter R1 | TTGGACAAAGTGGGTCTCCG |  |
| CHIP-LEF-1*-pten-* promter F2 | CCTGAAAGTAGTTCCGACCGC |  |
| CHIP-LEF-1*-pten-* promter R2 | GATCGCGATGGCCAGGTTC |  |

**Table S3: Primer sequence of the bisulfite-treated DNA utilized specific primers targeting CpG sites within the CpG islands of the LEF-1 gene core promoter.**

| Name | Sequence |
| --- | --- |
| LEF-1 promoter -S1-F | GTTATCGGTAGTTTTTTGTTTTGAT |
| LEF-1 promoter -S1-R | ATCCACTTCCTTCAAAATAAACAAT |

**Table S4. The binding of MBD2 to *Lef-1* sites and of LEF-1 to *Pten* probe sequence**

| Sites | Probe sequence |
| --- | --- |
| MBD2-LEF-1 GC01 | CGGGAACAAAGAGGGATCGGGCTCG |
| MBD2-LEF-1 GC02 | CGGGAACAAAGAGGGATCGGGCTCG |
| MBD2-LEF-1 GC03 | TTTGGGCCCGAAGCTCCTGCTG |
| MBD2-LEF-1 GC04 | CAAGACTCCGCC GTGCCAGCCA |
| MBD2-LEF-1 GC05 | CGCCGATTCCCAGCGCTCATCATCA |
| LEF-1- *Pten* Site 1 | GCGGCTCATGTATCCTTCCACCTCG**CCTTTGAG**CCCTCCCAGGCCTGCTCGCCCCGCCCACTC |
| LEF-1- *Pten* Site 2 and Site 3 | GGGTGGGGGCTTAGC**TCTTTGAA**GACTGAGCTTG**GCTGTGAT**CCGGTAGACCCACCGCTGCGG |

**(The positions designated for the primer are indicated in strikethrough,** **positions of the *Pten* promoter mutations are highlighted)**

**Table S5. Basic characteristics of SLE patients and healthy controls.**

| Sample | age | gender | SLEADI-2k | Sample | age | gender | SLEADI-2k |
| --- | --- | --- | --- | --- | --- | --- | --- |
| Patient 1 | 14 | Female | 5 | Patient 22 | 32 | Female | 2 |
| Patient 2 | 52 | Female | 4 | Patient 23 | 31 | Female | 2 |
| Patient 3 | 60 | Female | 0 | Patient 24 | 34 | Female | 7 |
| Patient 4 | 12 | Female | 3 | Patient 25 | 41 | Female | 2 |
| Patient 5 | 74 | Female | 5 | HC 1 | 28 | Female |  |
| Patient 6 | 40 | Female | 0 | HC 2 | 26 | Female |  |
| Patient 7 | 45 | Female | 3 | HC 3 | 23 | Female |  |
| Patient 8 | 49 | Female | 5 | HC 4 | 36 | Female |  |
| Patient 9 | 28 | Female | 3 | HC 5 | 34 | Female |  |
| Patient 10 | 18 | Female | 6 | HC 6 | 41 | Female |  |
| Patient 11 | 41 | Female | 3 | HC 7 | 28 | Female |  |
| Patient 12 | 49 | Female | 8 | HC 8 | 26 | Female |  |
| Patient 13 | 54 | Female | 2 | HC 9 | 23 | Female |  |
| Patient 14  Patient 16  Patient 17  Patient 18  Patient 19  Patient 20  Patient 21 | 30  32  30  45  55  32  31 | Female  Female  Female  Female  Female  Female  Female | 2  7  6  7  3  12  7 | HC 10  HC 11 | 36  32 | Female  Female |  |
