# Supplementary material for: MBD2 promotes B cell differentiation and BCR signaling in systemic lupus erythematosus by regulating the LEF-1-PTEN-PI3K axis
Source: Cell Death Dis. 2025 Jun 4;16(1):433. doi: 10.1038/s41419-025-07750-6 (PMC12137598; doi:10.1038/s41419-025-07750-6)
Supplement: Supplementary file 3 — Supplementary Figure2 [file 41419_2025_7750_MOESM3_ESM.pdf]

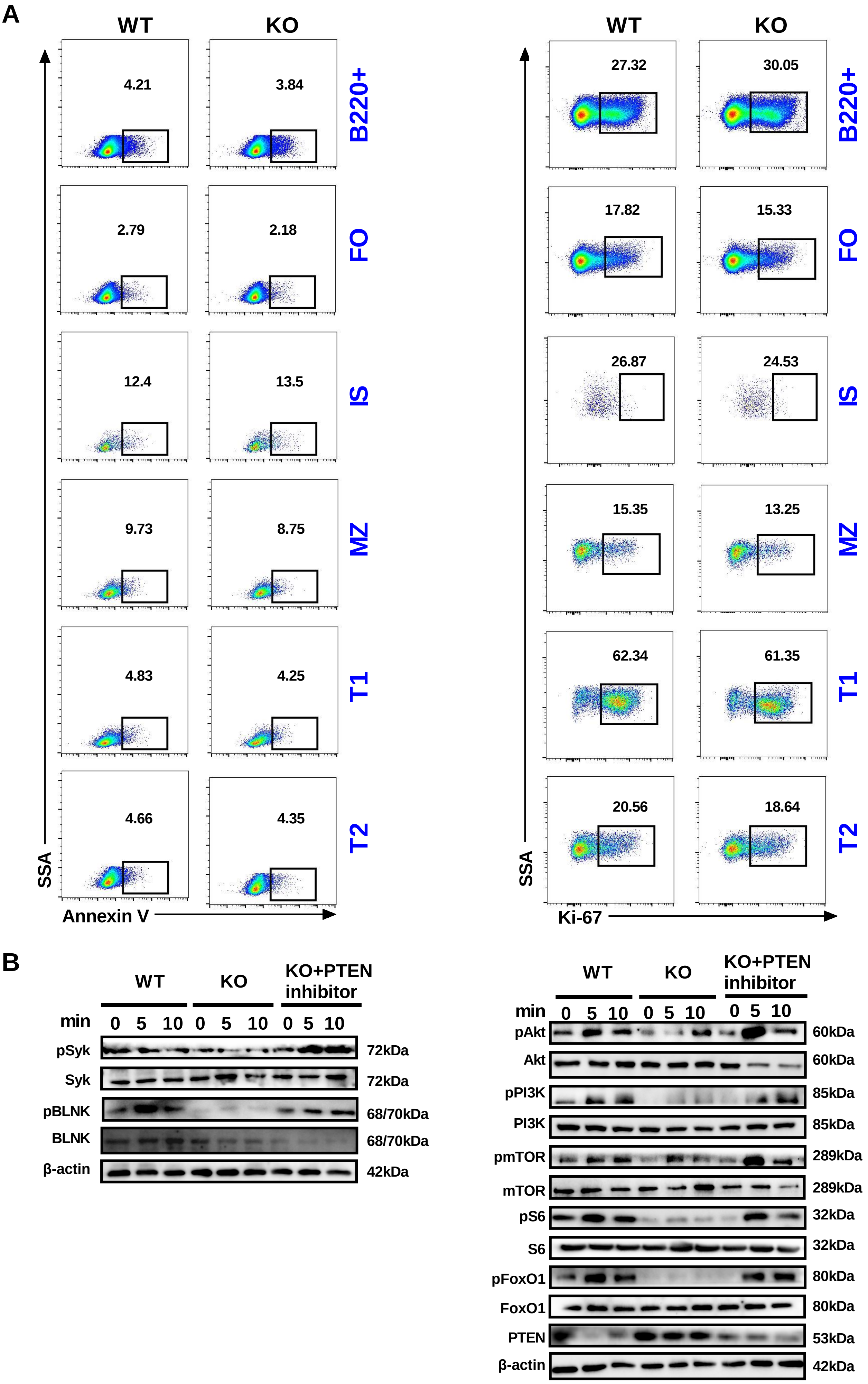
A
WT
KO
4.21
3.84
B220+
2.79
2.18
FO
12.4
13.5
IS
9.73
8.75
MZ
4.83
4.25
T1
4.66
4.35
T2
SSA
Annexin V
WT
KO
27.32
30.05
B220+
17.82
15.33
FO
26.87
24.53
IS
15.35
13.25
MZ
62.34
61.35
T1
20.56
18.64
T2
SSA
Ki-67
B
WT
KO
KO+PTEN inhibitor
min
0 5 10 0 5 10 0 5 10
pSyk
72kDa
Syk
72kDa
pBLNK
68/70kDa
BLNK
68/70kDa
β-actin
42kDa
WT
KO
KO+PTEN inhibitor
min
0 5 10 0 5 10 0 5 10
pAkt
60kDa
Akt
60kDa
pPI3K
85kDa
PI3K
85kDa
pmTOR
289kDa
mTOR
289kDa
pS6
32kDa
S6
32kDa
pFoxO1
80kDa
FoxO1
80kDa
PTEN
53kDa
β-actin
42kDa
